# Metabolic modeling with reverse mapping links host perturbations to *Mycobacterium tuberculosis*

**DOI:** 10.1016/j.isci.2026.117477

**Published:** 2026-09-17

**Authors:** Jayendrajyoti Kundu, Vishawjeet Barik, Abhijit Paul, Ankit Biswas, Pallavi Mudgal, Yashwant Kumar, Tushar Kanti Maiti, Amit Kumar Pandey, Samrat Chatterjee

**Affiliations:** 1 Complex Analysis Group, Computational and Mathematical Biology Centre, Translational Health Science and Technology Institute, NCR Biotech Science Cluster, Faridabad-Gurgaon Expressway, Faridabad 121001, India; 2 Mycobacterial Pathogenesis Laboratory, Centre for Tuberculosis Research, Translational Health Science and Technology Institute, NCR Biotech Science Cluster, Faridabad-Gurgaon Expressway, Faridabad 121001, India; 3 Turku Bioscience Centre, University of Turku and Åbo Akademi University, Turku, Finland; 4 Biomarker Discovery Laboratory, Translational Health Science and Technology Institute, NCR Biotech Science Cluster, Faridabad-Gurgaon Expressway, Faridabad 121001, India; 5 Laboratory of Functional Proteomics, Regional Centre for Biotechnology, NCR Biotech Science Cluster, Faridabad-Gurgaon Expressway, Faridabad 121001, India

**Keywords:** tuberculosis, host metabolic perturbation, genome scale metabolic model, host-pathogen interaction, CRISPRi, knockdown, omics data

## Abstract

Understanding host-pathogen interactions during *Mycobacterium tuberculosis* (Mtb) infection has largely relied on identifying bacterial genes essential for intracellular survival. However, conventional reverse genetics approaches often overlook host pathways that regulate Mtb growth. Here, we developed a backtracing strategy to link host metabolic modulators with specific Mtb proteins. A genome-scale host metabolic model integrated with lung transcriptomic data from H37Rv-infected mice predicted 18 host proteins essential for Mtb survival. We validated this through pharmacological inhibition, showing that the identified host metabolic regulators reduce the intracellular survival of wild-type H37Rv. Next, we constructed a host-pathogen protein interaction network that linked them to 9 Mtb proteins. Among them, *mce3E*, *fadA2*, and *ptpA* were identified through knockdown (KD) experiments as host-response modulators that favor pathogen growth. Proteomic and metabolomic analyses revealed their underlying molecular mechanisms. This was evaluated experimentally by modulating the corresponding host metabolic regulators, thereby restoring intracellular growth defects in Mtb KD strains and uncovering previously unrecognized host-pathogen survival mechanisms. Exogenous PGE2 treatment further suppressed intracellular bacterial survival.

## Highlights

• Developed a reverse-mapping framework linking host metabolism to Mtb effectors
• Predicted 18 host metabolic modulators of intracellular Mtb survival
• Experimentally validated host modulators and revealed new host-pathogen survival mechanisms
• Identified Mtb effectors and potential host-directed therapeutic targets for tuberculosis

## Graphical abstract

**Figure:**
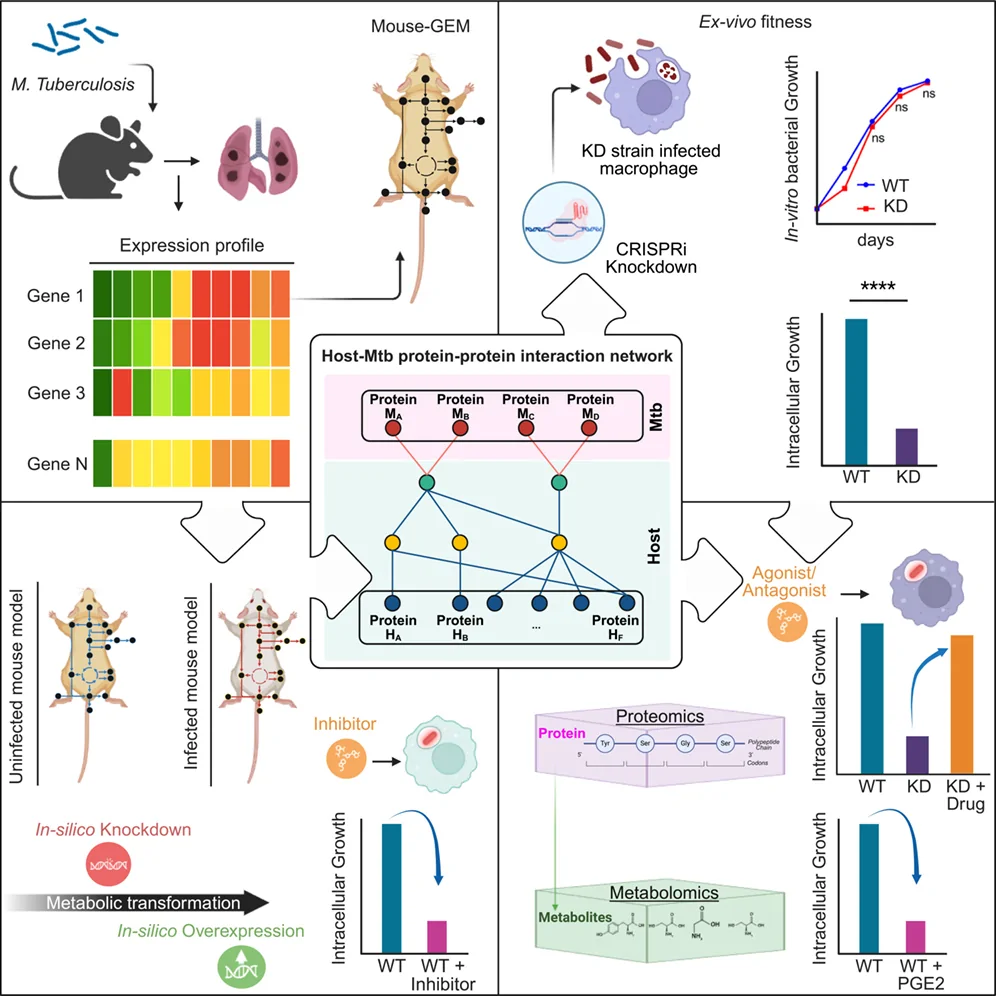

## Teaser

Immunology; Microbiology; Metabolomics

## Introduction

*Mycobacterium tuberculosis* (Mtb) is the causative agent of tuberculosis (TB), a leading global health threat responsible for millions of deaths annually.^1^ A key factor in its persistence is its ability to withstand hostile physiological conditions within host macrophages, enabling prolonged survival despite immune pressures.^2^ The current TB treatment regimen relies on multiple antibiotics for an extended duration, leading to toxicity and non-compliance-associated challenges.^3^ Given these challenges, elucidating the molecular mechanisms by which Mtb evades or manipulates host immunity is essential for developing novel therapeutic strategies that target host pathways. This host-directed therapy (HDT) approach will help enhance immune-mediated clearance of the pathogen, thereby potentiating the existing anti-TB regimen. Inhalation initiates the dissemination of tuberculosis and the subsequent engulfment of airborne Mtb by alveolar macrophages in the lungs.^4^ Depending on the immune status, this could lead to either an active infection with typical symptoms or a latent infection with no clinical manifestations. If left untreated, the pathogen could evade the host’s immune system and survive for an extended period inside the host. To do so, Mtb has evolved various strategies to evade the host defense mechanisms pivotal to clearing the pathogen and achieving a favorable disease outcome.^5^

Mtb possesses an arsenal of weapons that help neutralize the host-mediated insults essential for its survival inside the host. Mtb executes this by modulating various host pathways critical for restricting and neutralizing the pathogen, which mainly includes the host immune, cell signaling and metabolic-related pathways.^6^^,^^7^ Several Mtb secreted proteins, including PtpA, SapM, Ndk, and PknG, have been reported to actively inhibit phagosome maturation and block phagolysosome fusion by directly interacting with key host proteins.^8^^,^^9^^,^^10^^,^^11^^,^^12^ In addition to inhibiting these intracellular pathways, Mtb also modulates macrophage apoptosis at different stages of infection.^13^ During early infection, PtpA, a secretory protein of Mtb, dephosphorylates GSK3 to disrupt apoptotic signaling.^14^^,^^15^ Another Mtb-secreted protein, LpqN, interacts with the human E3 ubiquitin ligase CBL, which acts as a host restriction factor, to limit bacterial proliferation within macrophages.^16^ In parallel with these immune evasion strategies, Mtb extensively reprograms host metabolism to establish a nutrient-rich intracellular niche. Further, to sustain prolonged intracellular survival capabilities, Mtb induces lipid droplet accumulation in macrophages while simultaneously modulating key metabolic pathways, including glycolysis, oxidative phosphorylation, and lipid metabolism.^17^^,^^18^^,^^19^^,^^20^ Collectively, these findings highlight the multifaceted mechanisms employed by Mtb to evade host intrinsic immunity and reshape cellular metabolism, underscoring potential avenues for host-targeted TB interventions. In an era of increasing antibiotic resistance, mechanistic understanding and targeting of critical host-pathogen interaction pathways are high-priority research domains. Information from such studies is central to the development of host-based strategies against TB.^19^ While the host-pathogen interaction landscape in tuberculosis is flooded with studies examining the roles of individual Mtb proteins in mycobacterial pathogenesis, genome-scale analyses that involve both the pathogen and the host are very scarce. Nonetheless, using high-throughput unbiased genome-wide screening approaches, recent findings have systematically delineated complex host-pathogen interactions and have helped identify host perturbations that modulate bacterial growth and pathogenesis within the cell.^21^^,^^22^^,^^23^^,^^24^ Recent advances involve the use of CRISPR-based tools to validate the dual RNA-seq data, which led to the identification of the GID/CTLH complex critical for restricting the growth of Mtb inside the host.^25^

**Figure 1:**
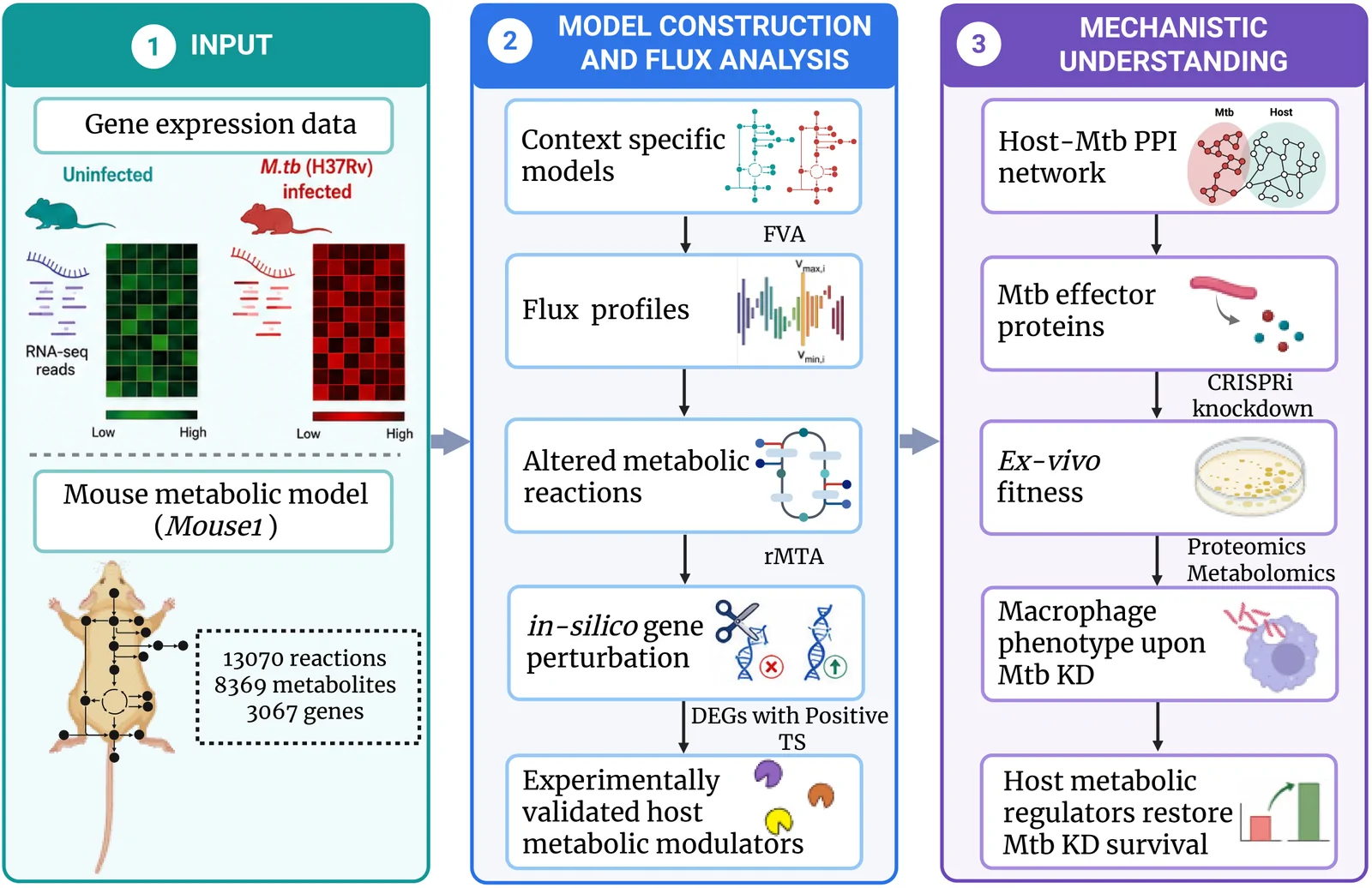
Schematic diagram of complete analytical framework The pipeline comprises three interconnected stages. **(1) Input:** RNA-seq data from uninfected and H37Rv-infected mouse lungs were integrated with the generic mouse genome-scale metabolic model (*Mouse1*) to reconstruct condition-specific metabolic models. **(2) Model construction and flux analysis:** Condition-specific models were generated using tINIT and E-flux. Flux variability analysis (FVA) identified flux profiles and altered metabolic reactions, followed by *in-silico* gene perturbation using rMTA to prioritize host metabolic modulators. Genes with consistently positive transformation scores (TS) were intersected with differentially expressed genes (DEGs) to identify host metabolic modulators associated with infection-induced metabolic reprogramming. **(3) Mechanistic understanding:** Identified host metabolic modulators were linked to secreted Mtb effector proteins through a literature-curated host-Mtb protein-protein interaction (PPI) network. Selected Mtb effectors were validated using CRISPRi knockdown strains, followed by *ex vivo* fitness and macrophage phenotypic analyses through targeted proteomics and untargeted metabolomics. Pharmacological modulation of the predicted host metabolic modulators restored the intracellular survival defects of Mtb knockdown strains, supporting the inferred host-pathogen interactions and their potential as host-directed therapeutic targets. The figure was created with BioRender.

Conventional reverse genetic approaches involving the generation of clean gene deletion strains of the pathogen to study host perturbations provide only a narrow view of the host microenvironment associated with specific pathogen perturbations. These approaches often fail to capture the full spectrum of host responses during infection. To overcome this limitation, we employed a backtracing strategy that links global host alterations to specific Mtb proteins. Such retrospective identification of host modulators and tracing them back to their upstream bacterial effectors remains underexplored and has received limited validation through reverse genetic approaches. In this study, we addressed this challenge by first computationally identifying metabolic genes that belong to host metabolic pathways and were differentially expressed in the lungs of mice infected with Mtb. We hypothesized that a subset of these differentially expressed host genes contributes to the formation of a niche that favors the growth of the pathogen within the host. Furthermore, we generated a host-pathogen interaction network and mined the available literature to functionally link the set of differentially expressed host genes to specific Mtb secretory proteins. Finally, proteomics and metabolomics analyses of macrophages infected with CRISPR-mediated knockdown strains for each of these Mtb proteins validated our *in-silico* findings by confirming the perturbations at the protein and metabolite levels and their implications on the growth of Mtb within macrophages. An overview of the complete analytical framework is provided in Figure 1.

## Results

### *In-silico* prediction for metabolic alterations in H37Rv-infected mouse

**Figure 2:**
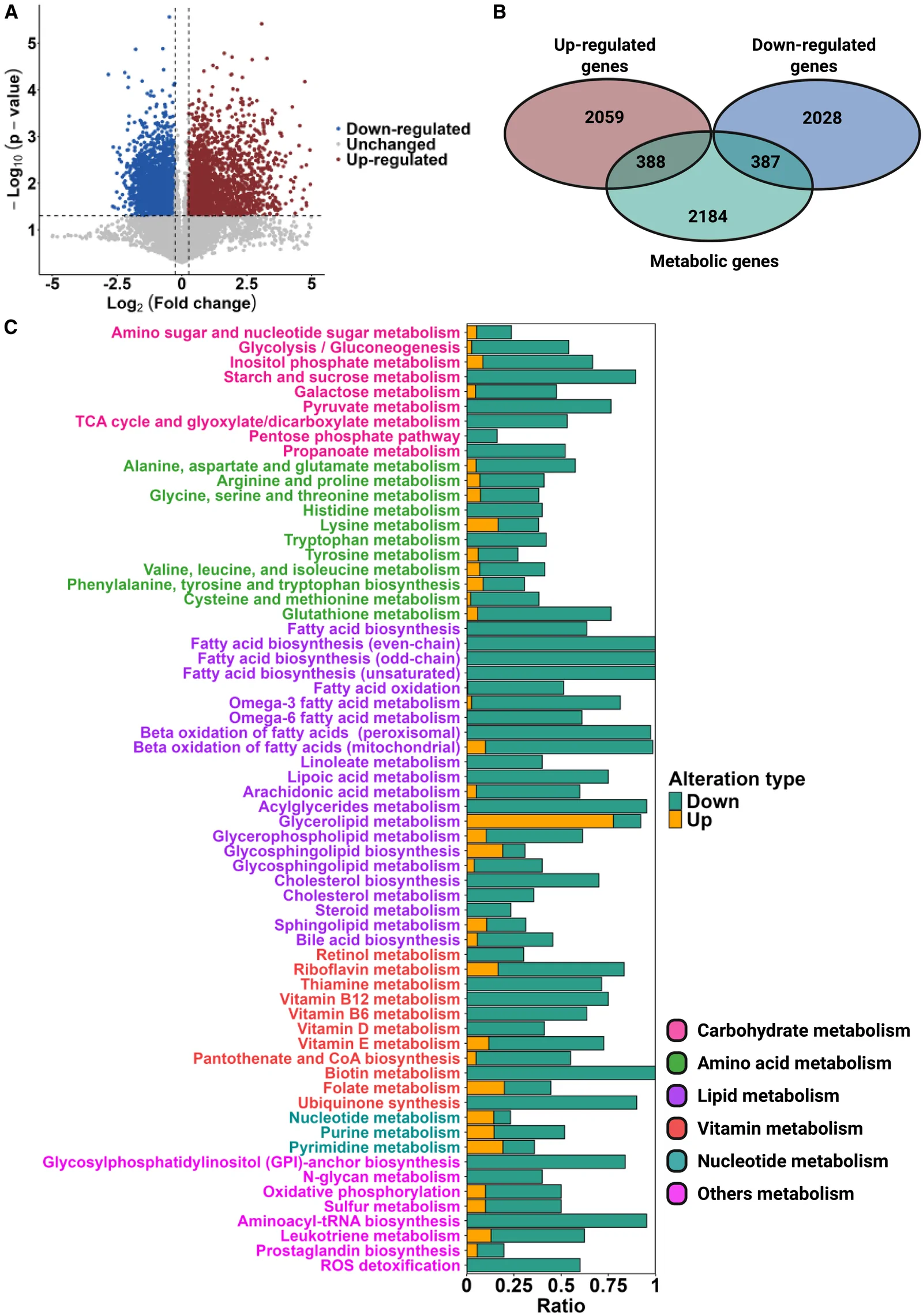
Metabolic profiles in H37Rv-infected mouse lungs (A) Volcano plot representation of differential expression analysis of RNA-seq data of Mtb-infected mouse lung tissue. DEGs were selected using the fold change cut-off value of 1.2 and *p* < 0.05. The up-and down-regulated genes are highlighted here in red and blue color, respectively. (B) Venn diagram of differentially expressed genes and metabolic genes. (C) Subsystems that include the altered metabolic reactions. The bar length represents the ratio of altered reactions found in each subsystem. Here, the subsystems were grouped according to their associated metabolic processes.

Long-term survival within the host requires Mtb to modulate host metabolic pathways to favor its survival. We analyzed the gene expression data from mice infected with Mtb and compared it with the uninfected group. Differentially expressed genes (DEGs) were calculated using a fold change cutoff of 1.2 with *p-*value <0.05 (Figure 2A). We obtained 2488 upregulated and 2415 downregulated genes (Table S1). Gene ontology analysis revealed that upregulated genes were enriched in processes related to autophagy, apoptosis, necrosis, phagocytosis, endoplasmic reticulum stress, inflammation, and nucleotide metabolism, and downregulated genes were enriched in lipid, carbohydrate, and amino acid metabolism (Figure S1A). We also observed that 775 metabolic genes are differentially expressed in the Mtb-infected group, with 388 upregulated and 387 downregulated (Figure 2B). For mechanistic understanding, we constructed contextualized metabolic models for H37Rv-infected and uninfected mice by integrating gene expression data with the generic mouse genome-scale metabolic model, Mouse1,^26^ using task-driven integrative network inference for tissues (tINIT)^27^ and the E-flux algorithms.^28^ The resulting models contained 7,750 and 7,869 active reactions, respectively, with 7,680 reactions shared between both (Figure S1B). These models were then analyzed to predict flux differences in Mtb-infected lung tissue. We observed 614 upregulated and 3574 downregulated reactions in the H37Rv-infected mouse model using a fold change cutoff of 1.2 (Table S2), spanning 125 subsystems within Mouse1, highlighting the significant alterations in host carbohydrate, amino acid, cholesterol, lipid, vitamin, nucleotide, and energy metabolism (Figure 2C). All pathways involved in carbohydrate metabolism were downregulated, consistent with previous findings that Mtb disrupts host glycolytic flux—particularly between fructose-6-phosphate and fructose-1,6-bisphosphate.^5^ Our data also confirmed downregulation of the TCA cycle and oxidative phosphorylation, in agreement with earlier reports.^29^ Host lipid metabolism was also significantly altered, with most reactions in glycerolipid metabolism upregulated, whereas linoleate, lipoic acid, acylglyceride, and steroid metabolism were downregulated. Additionally, marked alterations were observed in the biosynthesis of even-chain, odd-chain, and unsaturated fatty acids as well as omega-6 fatty acid metabolism and fatty acid beta-oxidation.

### Genes responsible for the metabolic alterations

**Figure 3:**
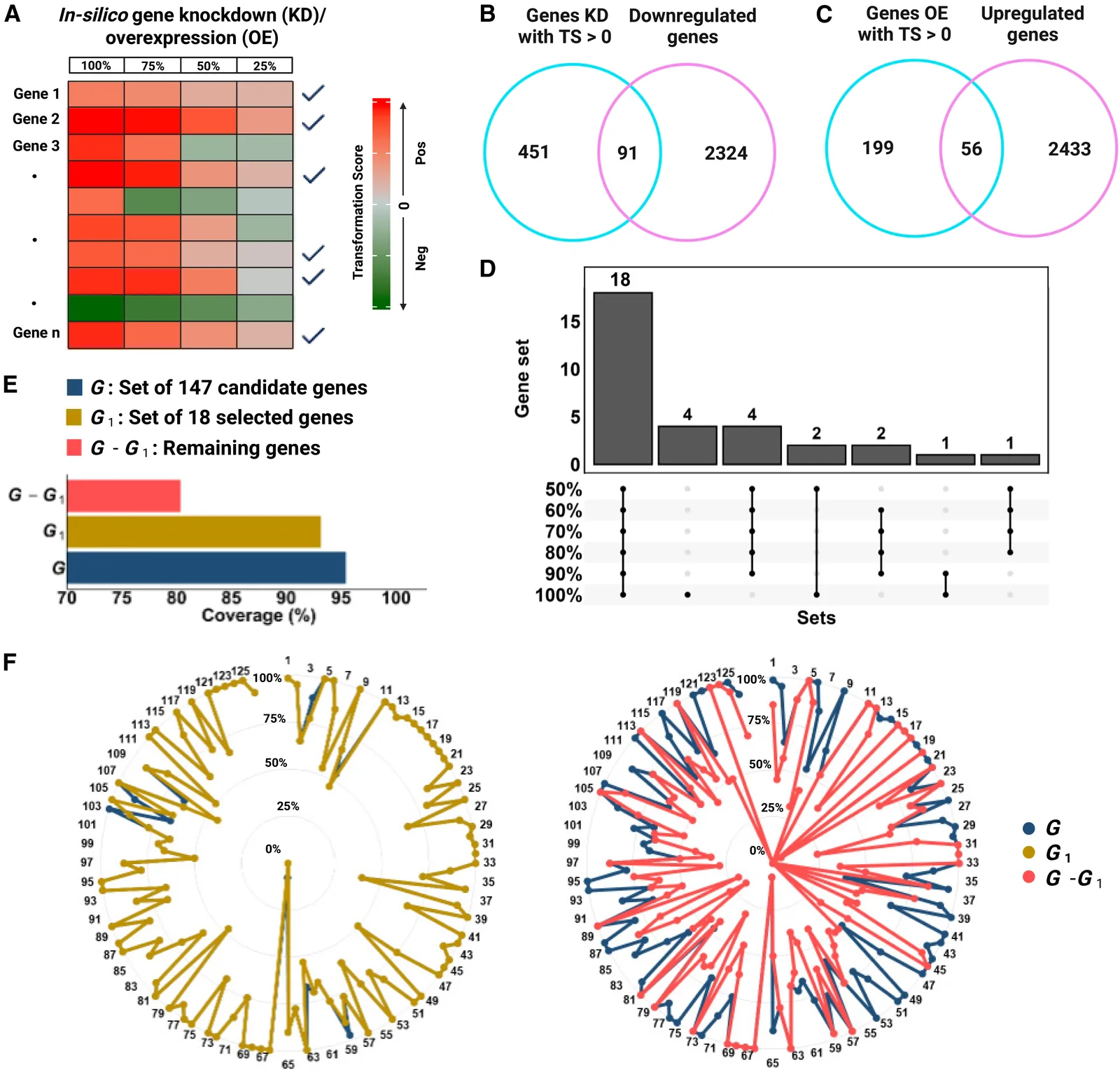
Metabolic transformation analysis (A) Schematic diagram of *in-silico* gene knockdown (KD) and overexpression (OE) strategy. (B) Venn diagram of KD genes with consistent positive transformation scores (TS) and down-regulated genes. (C) Venn diagram of OE genes with consistent positive TS and upregulated genes. (D) Upset plot between the top 25 gene sets for each threshold *x%*, where *x* = 50, 60, …, 100. (E) The bar plot represents the collective percentage coverage of all observed altered reactions in the H37Rv-infected mouse model by the gene sets *G*, *G*_1_, and *G* − *G*_1_. (F) The radar plot represents the collective percentage coverage of altered metabolic reactions for each of the 125 metabolic subsystems covered by the gene sets *G*, *G*_1_, and *G* − *G*_1_. The names of these 125 metabolic subsystems are given in Table S4.

We look for host genes responsible for shifting the host metabolic signature from the uninfected toward the H37Rv-infected state. Such insights could help identify critical host pathways that the pathogen specifically modulates to establish an intracellular niche that favors its growth within the cell. To investigate this, we first performed *in-silico* knockdown and overexpression analyses of host metabolic genes and assessed the consequences. Genes exhibiting consistently positive robust transformation scores (rTS) across different perturbation levels (25–100% with 25% increment) were selected for downstream analysis (Figure 3A; Table S3). Knockdown of 542 genes met this criterion (rTS>0 for all knockdown percentages), of which 91 were also found to be downregulated in H37Rv-infected mice (Figure 3B). Similarly, overexpression of 255 genes satisfied the criterion, with 56 of them also upregulated in H37Rv-infected mice (Figures 3C; Table S3). This resulted in a total of 147 candidate host metabolic genes. Collectively, perturbations in these 147 genes accounted for 95.4% of the altered metabolic reactions observed in the H37Rv-infected mouse model (Figure 3E).

To enable a more targeted investigation, we aimed to identify a smaller subset of genes whose modulation could collectively explain most of the observed changes across the 125 affected metabolic subsystems. Using the subsystem count ratio (*R*_*k*_(*x*)) and reaction coverage (*C*_*k*_) as selection criteria, we identified a subset of 18 genes (*G*_1_) that collectively captured nearly all altered metabolic reactions (Figure 3D, STAR Methods). These 18 genes accounted for 93.1% of the altered reactions, compared to 80.4% covered by the remaining genes (Figure 3E). Further analysis revealed that perturbations in these 18 genes provided nearly equivalent subsystem-level coverage to the whole gene set across most affected metabolic subsystems (Figure 3F). In contrast, collective perturbations in the remaining genes failed to achieve comparable coverage. We also performed hierarchical clustering of all 147 perturbed genes based on their percentage coverage of altered reactions across the 125 subsystems. The 18 selected genes consistently ranked highest (Figure S2A), exhibited the greatest overall coverage of altered reactions (Figure S2B), and achieved top ranks in terms of the number of subsystems with maximal percentage coverage (Figure S2B).

**Figure 4:**
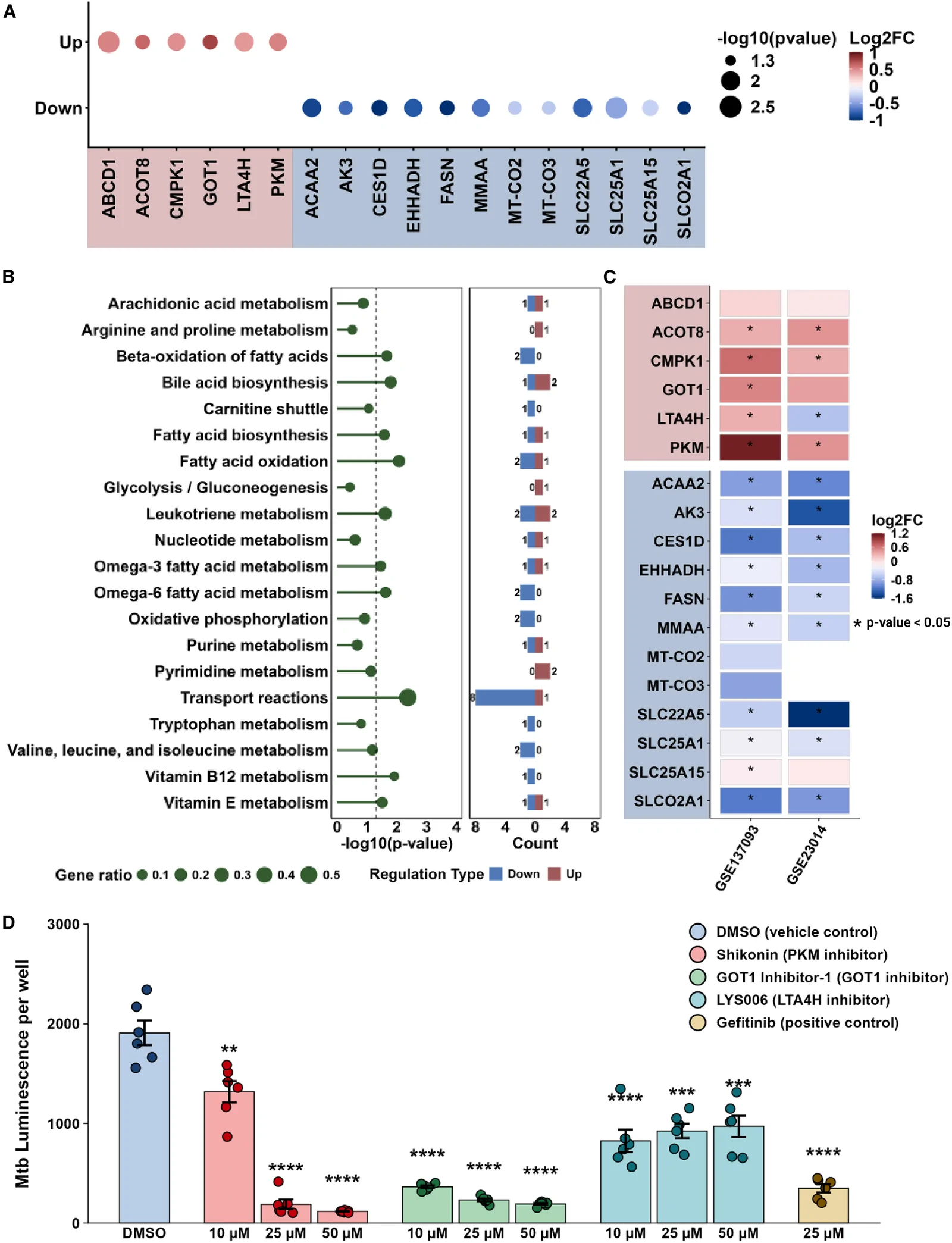
Host genes whose perturbation leads to the metabolic perturbation (A) Bubble plot represents the fold change of the selected 18 genes (*G*_1_). Here, the color and size of the bubble represent the fold change (on a log2 scale) and the *p-*value (on a -log10 scale), respectively. (B) Subsystems enriched by this gene set, examined by hypergeometric distribution. Here, the numbers of up- and down-regulated genes were plotted using red- and blue-coloured bars, respectively. (C) A heatmap showing the log2 fold changes (FC) in the expression patterns of these 18 genes in H37Rv-infected mouse lung tissue versus uninfected in two independent datasets: GSE137093 and GSE23014. Here, red, blue, and white denote upregulation, downregulation, and no change, respectively. Statistical significance was assessed using Student’s unpaired *t* test. ∗, *p* < 0.05. (D) THP-1 macrophages were infected with auto-bioluminescent H37Rv at a multiplicity of infection (MOI) of 10 bacteria per macrophage and treated with increasing concentrations (10, 25, and 50 *μ*M) of Shikonin (PKM inhibitor), GOT1 Inhibitor-1 (GOT1 inhibitor), or LYS006 (LTA4H inhibitor), with 0.05% DMSO as the vehicle control and Gefitinib (25 *μ*M) as the positive control. Intracellular bacterial survival was quantified by measuring luminescence on day 4 post-treatment. Each group included six biological replicates. Data are presented as mean ± SD from independent biological replicates. Statistical significance was determined using ordinary one-way ANOVAs with Dunnett’s multiple-comparison test, ratio paired *t* tests, or unpaired *t* tests. ∗*p* < 0.05, ∗∗*p* < 0.01, ∗∗∗*p* < 0.001, and ∗∗∗∗*p* < 0.0001; ns, not significant.

Among those 18 differentially expressed genes, 6 were upregulated, and 12 were downregulated in lung tissue infected with H37Rv relative to uninfected control (Figure 4A). Notably, all 18 genes showed distinct and statistically significant expression differences between infected and uninfected conditions (Figure S3). Pathway analysis revealed that this set of genes is enriched in pathways related to fatty acids, amino acids, bile acids, nucleotides, and vitamin metabolism (Figure 4B). Notably, most of these genes (9 out of 18) encode transporters, suggesting a potential shift in metabolite trafficking during infection. Given the limited number of replicates in the primary transcriptomic dataset used for metabolic model construction (three infected and two uninfected samples), we sought to independently validate the expression patterns of the 18 prioritized host genes in two additional publicly available mouse lung transcriptomic datasets: GSE137093^30^ (five infected and five uninfected) and GSE23014^31^ (five infected and three uninfected), to ensure the robustness and reproducibility of our findings. GSE137093 includes data from five H37Rv-infected and five uninfected C57BL/6 mice at 42 days post-infection, while GSE23014 comprises data from five infected and three uninfected biological replicates across four time points (0, 12, 15, and 21 days post-infection); for consistency, we used the latest available time point for comparison. Consistent expression patterns were observed for all of these genes across both datasets, except LTA4H in GSE23014 (Figures 4C, S4, and S5), suggesting a potential regulatory impact of Mtb infection on their expression. To further evaluate the therapeutic relevance of the identified host metabolic modulators, we investigated whether pharmacological targeting of these host factors could directly affect intracellular survival of wild-type H37Rv. Treatment of H37Rv-infected macrophages with Shikonin (PKM inhibitor), GOT1 Inhibitor-1 (GOT1 inhibitor), LYS006 (LTA4H inhibitor), or Gefitinib (positive control) significantly reduced intracellular bacterial survival compared with untreated infected macrophages (Figure 4D).

### Mtb proteins that drive the host metabolic perturbation

**Figure 5:**
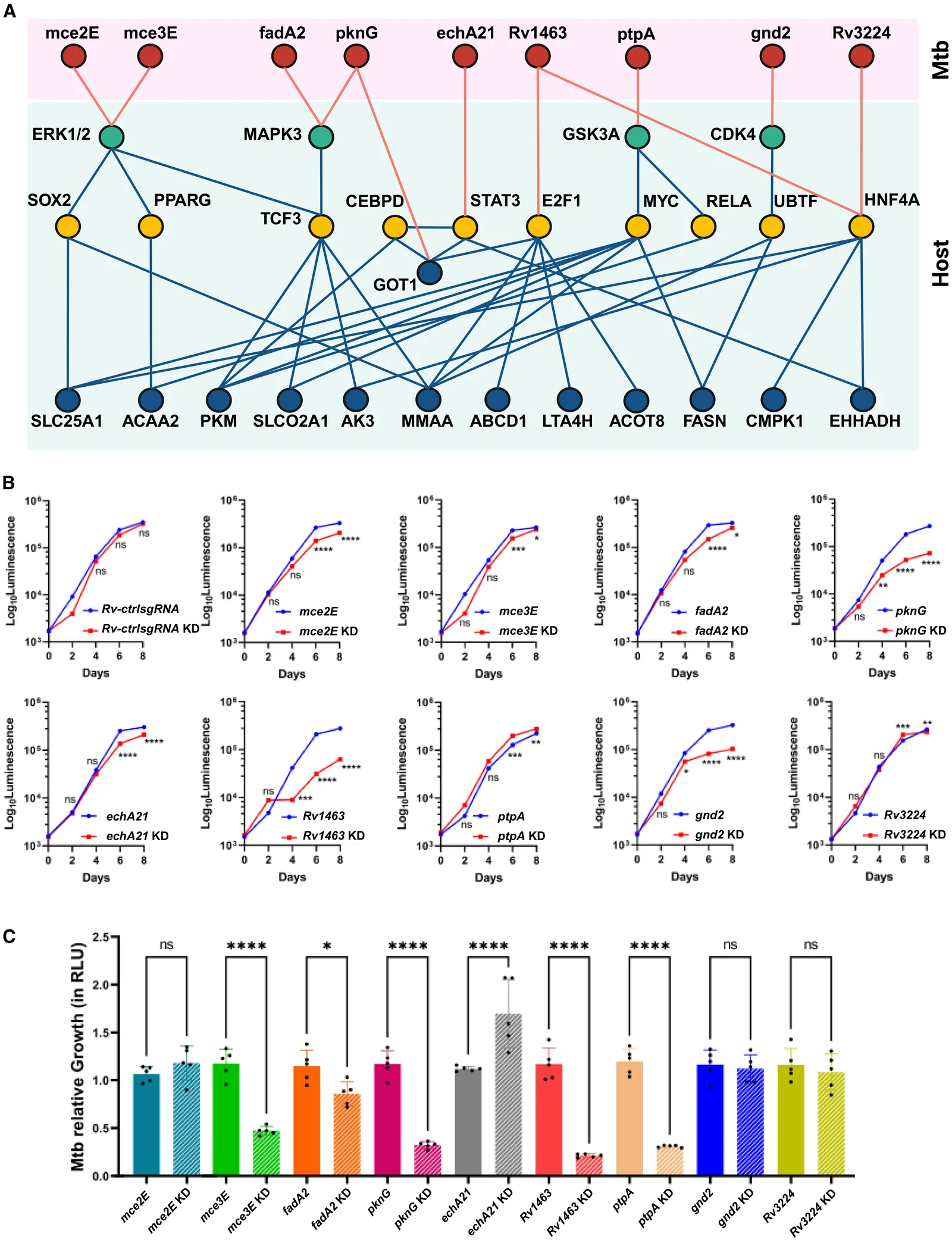
Mtb proteins interacting with host metabolic proteins and their role in the pathogen’s survival fitness (A) A minimal directed host-pathogen interaction network for selected host metabolic gene products. Secreted Mtb proteins are shown in red color. Host metabolic proteins that interact directly and indirectly with 9 Mtb proteins are shown in blue. Transcription factors and their first upstream interactors are shown in yellow and green colors, respectively. The orange edge line represents interactions between Mtb proteins and host proteins, while the blue edge line indicates interactions among host proteins. (B) *In vitro* growth kinetics of Mtb CRISPRi knockdown strains upon ATc (100 ng/mL) treatment. Statistical significance has been made using two-way ANOVA (Šídák’s multiple comparisons test) where ∗*p* = 0.0102, ∗∗*p* = 0.0043, ∗∗∗*p* = 0.0005, ∗∗∗∗*p* < 0.0001, and ns P>0.9999. (C) Intra-macrophage survival assay using THP-1 monocyte-derived macrophages. The relative growth of Mtb knockdown strains was determined using luminescence. Data are represented as the mean ± SEM of the replicates indicated by points in the graph. Statistical significance was assessed against the –ATc group using an ordinary one-way ANOVA (Holm-Šídák’s multiple comparisons test). ∗, *p* < 0.01; ∗∗∗∗*p* < 0.0001; ns, non-significant.

To understand the interplay between Mtb and host metabolic perturbation, we focused on identifying Mtb proteins that could modulate the expression or function of the 18 selected host metabolic genes. As a first step, we identified 99 host transcription factors (TFs) that regulate the expression of these genes (Table S5). We then sought to establish connections between Mtb proteins and these TFs. Through literature mining of host-pathogen interactions^32^^,^^33^^,^^34^^,^^35^^,^^36^^,^^37^^,^^38^^,^^39^^,^^40^^,^^41^ (see STAR Methods, Table 1), we identified direct interactions between the Mtb proteins Rv1463, Rv3224, and EchA21 and the host TFs E2F1, HNF4A, and STAT3, respectively. These interactions allowed us to link nine host metabolic genes to Mtb proteins. For the remaining genes, we identified the first upstream interactors of their respective TFs within a directed protein-protein interaction network. We investigated their potential interactions with Mtb proteins based on evidence from the literature. Using this approach, we linked four additional host metabolic genes to Mtb proteins via four upstream interactors of their associated TFs (ERK1/2, MAPK3, GSK3A, and CDK4). Finally, we obtained a minimal directed host-pathogen interaction network comprising 13 host metabolic gene products and nine Mtb proteins (Figure 5A). It was also observed that a secreted Mtb protein, PknG, directly interacts with the GOT1 protein product,^33^ a member of this 13-gene set. These findings suggest that these nine Mtb proteins may significantly modulate host metabolism through this network, warranting further experimental validation.

**Table 1:** Literature-curated host-pathogen interaction dataset used for construction of the host-pathogen interaction network

Mtb protein	Evidence for secretion/EV localization	Host interactor	Reported interaction
mce2E	secreted protein^34^^,^^41^	ERK1/2	directly binds ERK1/2 and inhibits its activation.^34^^,^^41^
mce3E	secreted protein^41^	ERK1/2	directly binds ERK1/2 and inhibits its activation.^41^
fadA2	extracellular vesicle-associated^42^	MAPK3	reported host-pathogen interaction.^37^
pknG	secreted protein^43^^,^^44^	MAPK3	reported host-pathogen interaction.^33^
echA21	extracellular vesicle-associated^42^	GOT1	reported host-pathogen interaction.^33^
echA21	extracellular vesicle-associated^42^	STAT3	reported host-pathogen interaction.^39^
Rv1463	secreted protein^45^	E2F1	reported host-pathogen interaction.^39^
Rv1463	secreted protein^45^	HNF4A	reported host-pathogen interaction.^39^
ptpA	secreted protein^46^	GSK3A	directly dephosphorylates gsk3a.^14^
gnd2	extracellular vesicle-associated^42^	CDK4	reported host-pathogen interaction.^39^
Rv3224	extracellular vesicle-associated^42^	HNF4A	reported host-pathogen interaction.^39^

To investigate the role of the nine Mtb proteins predicted to modulate host metabolism and promote pathogen survival, we generated CRISPRi-based knockdown (KD) strains of H37Rv targeting each of these genes. These KD strains exhibited anhydrotetracycline (ATc)-dependent depletion of the target genes (Figure S6). Using a luminescence-based assay, we then compared the survival fitness for all these KD strains with and without ATc conditions. We found that Mtb strains lacking *pknG*, *Rv1463,* and *gnd2* genes demonstrated an *in vitro* growth defect phenotype on 7H9-based enriched media (Figure 5B). Whereas, *ex vivo* growth assays revealed that Mtb strains lacking *mce3E*, *fadA2*, *pknG*, *Rv1463*, and *ptpA* had impaired survival within macrophages (Figure 5C). These findings indicate that *mce3E*, *fadA2*, and *ptpA* are non-essential for *in vitro* growth of Mtb but are conditionally essential for survival within macrophages, underscoring their potential role in modulating the host environment.

### Impact of Mtb knockdown strains on host metabolic proteins

Next, we sought to investigate the role of specific Mtb proteins in modulating host protein expression. To this end, we performed targeted proteomic analysis of macrophages infected with Mtb knockdown strains, each deficient in a specific protein. Based on the significant phenotypic changes observed in the survival fitness results, three Mtb proteins: Mce3E, FadA2, and PtpA, were selected, and their associated host metabolic proteins were identified from Figure 5A. Mce3E was linked to six host metabolic proteins, FadA2 to four, and PtpA to six, with seven unique host metabolic proteins associated with at least one of these Mtb proteins. Among them, PKM was upregulated in wild-type *mce3E*-infected macrophages compared to uninfected controls, while the remaining proteins were downregulated, consistent with the transcriptomic data.

**Figure 6:**
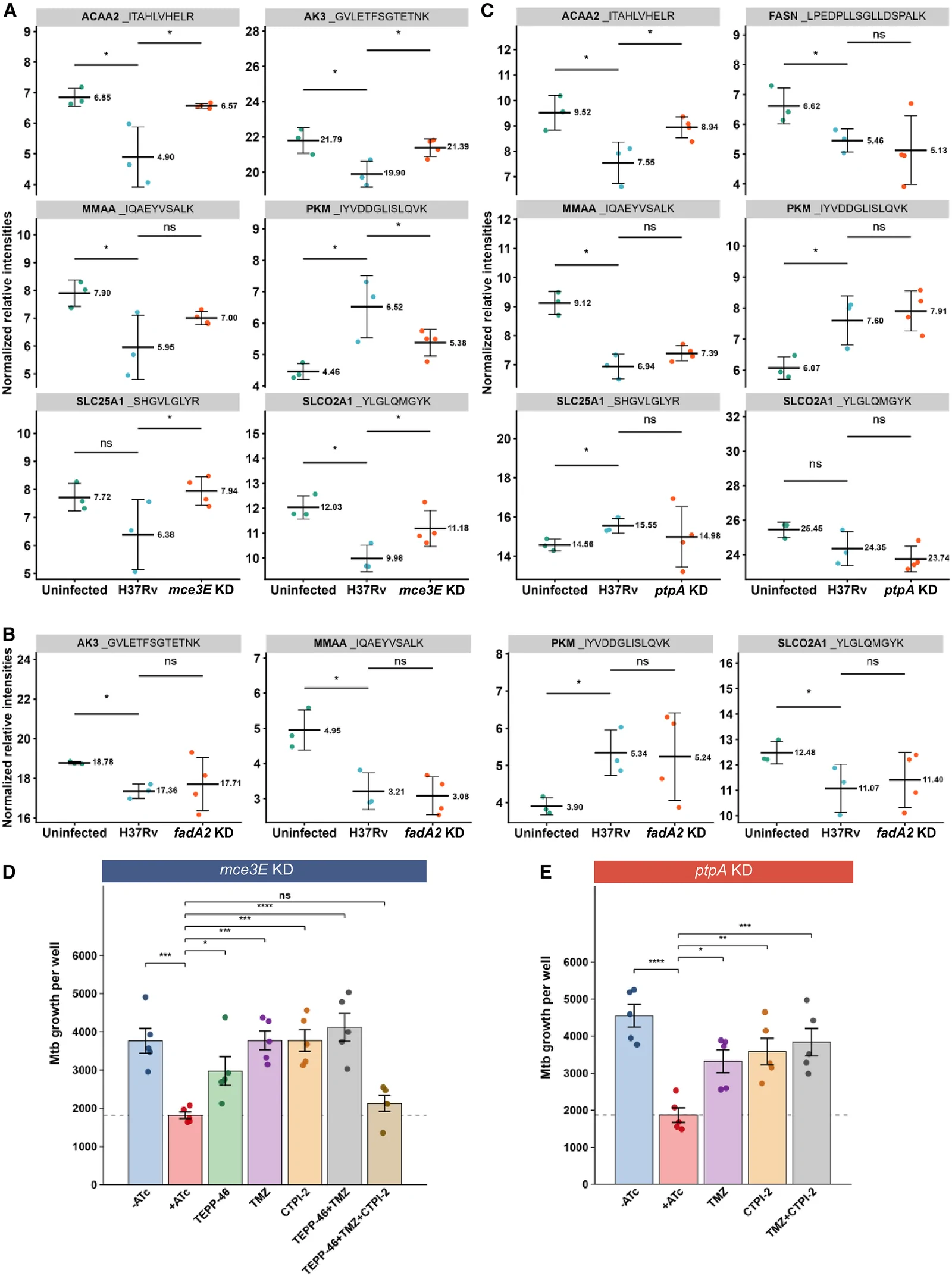
Comparison of host metabolic protein levels in macrophages infected with Mtb knockdown strains versus wild-type Normalized relative intensities were plotted for specific host metabolic proteins connected with the Mtb proteins (A) Mce3E, (B) FadA2, (C) PtpA. The corresponding host proteins were identified from Figure 5A. Each of the three H37Rv knockdown strains included three biological replicates under the -ATc (wild-type) condition and four under the +ATc (knockdown) condition, along with three biological replicates of uninfected samples. The expression data were sum-normalized, log10-transformed, and Pareto-scaled. Here, data are represented as mean ± SEM, with individual replicates indicated by data points. Protein abundance measured via targeted proteomics was represented by corresponding peptide abundances, as labeled above each plot. Statistical analysis was performed using Student’s unpaired *t* test between two groups, where ∗, *p* < 0.05; ns, non-significant. (D and E) For growth rescue, THP-1 macrophages were infected with auto-bioluminescent *mce3E* or *ptpA* KD strains at a multiplicity of infection (MOI) of 2 bacteria per macrophage and treated with the indicated pharmacological modulators at a concentration of 25 *μ*M. For the *mce3E* KD strain, TEPP-46 (PKM2 activator), TMZ (ACAA2 inhibitor), CTPI-2 (SLC25A1 inhibitor), and the indicated combinations were used, whereas the *ptpA* KD strain was treated with TMZ, CTPI-2, or their combination. Anhydrotetracycline (ATc; 300 ng/mL) was used to induce gene knockdown, and 0.05% DMSO served as the vehicle control. Intracellular bacterial survival was quantified by measuring luminescence on day 5 post-treatment. Each group included five biological replicates. Data are presented as mean ± SD from independent biological replicates. Statistical significance was determined using ordinary one-way ANOVAs with Dunnett’s multiple-comparison test, ratio paired *t* tests, or unpaired *t* tests. ∗*p* < 0.05, ∗∗*p* < 0.01, ∗∗∗*p* < 0.001, and ∗∗∗∗*p* < 0.0001; ns, not significant.

By comparing the targeted proteomics data of these KD strains with the wild-type, we observed opposite expression patterns for most of these host metabolic proteins in at least one knockdown condition (Figure 6). The most pronounced effect was seen in the *mce3E* KD, which resulted in reversed expression trends for all of its linked host metabolic proteins (Figure 6A). However, knockdown of *fadA2* and *ptpA* led to reversed expression patterns in two out of four and two out of six linked host metabolic proteins, respectively, although some of these changes did not reach statistical significance (Figures 6B and 6C).

Based on the observed changes in host metabolic regulators, we next investigated whether pharmacological targeting of the predicted host metabolic regulators could restore the intracellular growth defects of the corresponding Mtb KD strains. Treatment of macrophages infected with the *mce3E* KD strain using TEPP-46 (PKM activator), TMZ (ACAA2 inhibitor), CTPI-2 (SLC25A1 inhibitor), or the combination of TEPP-46 and TMZ significantly restored intracellular bacterial growth compared with the untreated KD strain (Figure 6D). In contrast, simultaneous treatment with all three compounds did not produce additional restoration. Similarly, treatment of the *ptpA* KD strain with TMZ, CTPI-2, or their combination significantly restored intracellular bacterial growth relative to the untreated KD strain (Figure 6E).

### Metabolic profile of macrophages upon knockdown strains

**Figure 7:**
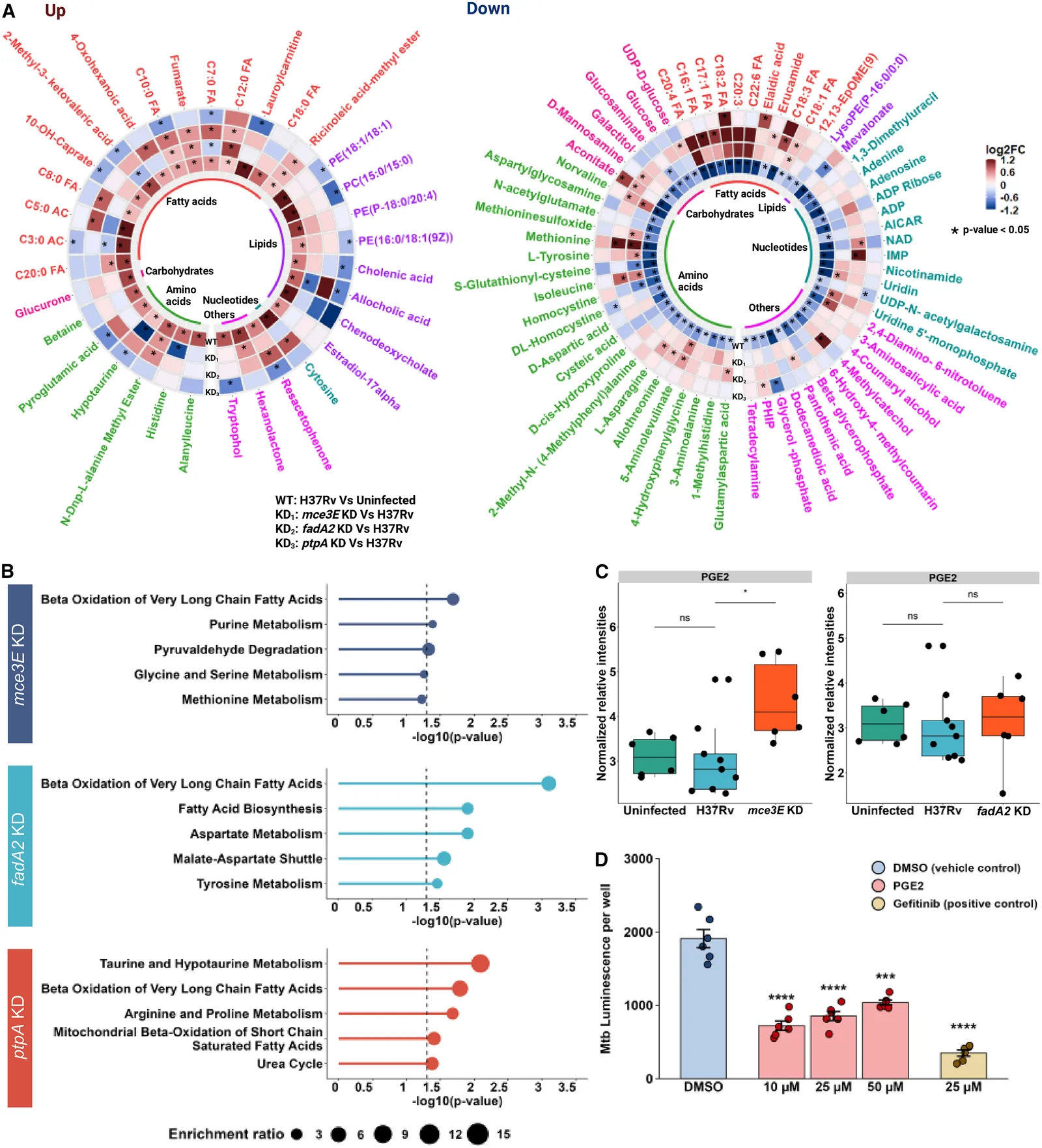
Comparison of metabolomics profiles of THP-1 cells infected with Mtb knockdown strains versus wild-type (A) The left and right panels depict circular heatmaps showing fold changes of upregulated and downregulated metabolites, respectively, in wild-type H37Rv-infected THP-1 macrophages compared to uninfected controls, along with their fold changes in three different KD strains compared to wild-type. The circular rows represent the following comparisons: WT: wild-type H37Rv vs. Uninfected, KD_1_: *mce3E* KD vs. wild-type, KD_2_: *fadA2* KD vs. wild-type, and KD_3_: *ptpA* KD vs. wild-type. Each of the three H37Rv knockdown strains under the -ATc (knockdown) condition included six biological replicates, along with nine biological replicates of the wild-type condition and six biological replicates of uninfected controls. The metabolic data were normalized by sum, log10-transformed, and scaled using the Pareto-scaling feature. The fold change threshold was 1.2 with a *p* < 0.05. (B) Pathways enriched by differentially expressed metabolites in *mce3E* KD, *fadA2* KD, and *ptpA* KD strains compared to wild-type H37Rv-infected macrophages. An over-representation test was performed to obtain the enriched pathways. Results are depicted in red, sky blue, and dark blue colored lollipop plots, respectively, with bubble sizes indicating the enrichment ratio. (C) Boxplot represents the level of PGE2 in *mce3E* and *fadA2* KD strain-infected macrophages as well as wild-type H37Rv-infected and uninfected macrophages. Statistical analysis was performed using Student’s unpaired *t* test between two groups, where ∗, *p* < 0.05; ns, non-significant. (D) THP-1 macrophages were infected with auto-bioluminescent H37Rv at a multiplicity of infection (MOI) of 10 bacteria per macrophage and treated with increasing concentrations (10, 25, and 50 *μ*M) of PGE2, with 0.05% DMSO as the vehicle control. Intracellular bacterial survival was quantified by measuring luminescence on day 4 post-treatment. Each group included six biological replicates. Data are presented as mean ± SD from independent biological replicates. Statistical significance was determined using ordinary one-way ANOVAs with Dunnett’s multiple-comparison test, ratio paired *t* tests, or unpaired *t* tests. ∗*p* < 0.05, ∗∗*p* < 0.01, ∗∗∗*p* < 0.001, and ∗∗∗∗*p* < 0.0001; ns, not significant.

To investigate the impact of Mtb proteins on the host metabolome, we performed untargeted metabolomics on THP-1 macrophages infected with three different CRISPRi-mediated knockdown strains targeting *ptpA*, *fadA2*, and *mce3E* genes of Mtb. Uninfected THP-1 cells were used as a control. In total, we identified 323 different metabolites. We first compared the change in the metabolome of wild-type infected cells to that of uninfected cells. This was followed by a comparative analysis of differences in the metabolic signature of the macrophages infected with wild-type and different knockdown strains. This analysis led us to identify distinct metabolic alterations driven by specific Mtb proteins. In total, 96 altered metabolites were identified in H37Rv-infected cells, of which 33 were upregulated and 63 were downregulated (Figure 7A). Notably, PE (18:1/18:1), PC (15:0/15:0), PE (P-18:0/20:4), and PE (16:0/18:1(9Z)) were found to be upregulated in H37Rv-infected cells. These findings are consistent with our metabolic model predictions, which indicated increased metabolic flux through the glycerophospholipid metabolism pathway, suggesting that Mtb may actively modulate this pathway to support its survival or replication. This observation aligns with previous studies reporting that Mtb infection significantly alters host lipid metabolism, including elevated levels of various phospholipids within infected macrophages.^47^^,^^48^ In contrast, concentrations of dodecanedioic acid and adenine were reduced in H37Rv-infected macrophages, correlating with model-predicted decreases in the flux of reactions involved in their production. Additionally, various fatty acids and lipids were elevated in H37Rv-infected THP-1 cells, whereas most amino acids, nucleotides, and carbohydrates were significantly downregulated. To further explore the global metabolic impact, reporter pathway analysis was performed, revealing several significantly altered pathways in H37Rv-infected THP-1 cells (Figure S7A), most of which were consistent with the altered subsystems predicted by our metabolic models (Figure 2C). Next, we compared the metabolomic profiles of three knockdown strains to those of the wild-type H37Rv strain. We observed opposite patterns for most of these altered metabolites (Figure 7A), indicating their significant role in host metabolic perturbation during Mtb infection. Of the 96 altered metabolites, 91 exhibited opposite trends in at least one knockdown strain. Specifically, the *mce3E*, *fadA2,* and *ptpA* KD strains showed reversed trends in 56, 60, and 62 metabolites, respectively. Furthermore, our analysis revealed that more than 50% reversed patterns were observed for 22, 32, and 20 metabolites in the macrophages infected with the *mce3E*, *fadA2,* and *ptpA* KD strains, respectively (Figures S7B–S7D).

Compared to the wild-type H37Rv strain, the *mce3E* KD strain exhibited 56 upregulated and 34 downregulated metabolites; the *fadA2* KD strain showed 71 upregulated and 3 downregulated metabolites; and the *ptpA* KD strain had 11 upregulated and 29 downregulated metabolites (Figures S7E–S7G). In macrophages infected with the *mce3E* KD strain, differentially expressed metabolites were significantly enriched in the beta-oxidation of long-chain fatty acids and purine metabolism pathways (Figure 7B), consistent with increased protein levels of ACAA2 and AK3, key enzymes in these pathways. Similarly, enrichment of the beta-oxidation of long-chain fatty acids and mitochondrial beta-oxidation of short-chain saturated fatty acids pathways was observed in the *ptpA* KD strain, correlating with upregulation of ACAA2, a key protein in this process. The mitochondrial citrate carrier SLC25A1, a key regulator of prostaglandin E2 (PGE2) production in macrophages,^49^ was upregulated in the *mce3E* KD strain compared to the wild-type strain. Additionally, the prostaglandin transporter SLCO2A1, which facilitates PGE2 influx into the cytosol,^50^ was upregulated in *mce3E* and *fadA2* KD strains. As a consequence, macrophages infected with these two KD strains exhibited elevated PGE2 levels compared to wild-type (Figure 7C). Consistent with the elevated PGE2 levels observed in the KD strains, exogenous PGE2 treatment significantly reduced the intracellular survival of wild-type H37Rv in infected macrophages (Figure 7D).

## Discussion

Decades of research have established that Mtb infection triggers a complex and dynamic interplay between the pathogen and the host metabolic environment. Mtb infection is associated with extensive remodeling of host metabolic pathways that is thought to facilitate intracellular survival. However, the multidimensional complexity introduces additional challenges in experimentally delineating the full extent of host metabolic perturbation. Continually refining novel host-directed therapy (HDT) approaches warrants a detailed mechanistic understanding of how critical Mtb secretory proteins interact and modulate host metabolic networks. In this study, we analyzed the differentially expressed genes in the lung tissue of mice infected with Mtb. The host gene expression data were integrated into Mouse1,^26^ a comprehensive genome-scale model of mouse metabolism, enabling a systems-level view of infection-driven metabolic rewiring. Constraint-based metabolic modeling predicted widespread suppression of metabolic activity, with notable reduction in flux through glycolysis, the tricarboxylic acid (TCA) cycle, oxidative phosphorylation, and fatty acid metabolism. These findings align with previous reports suggesting that Mtb induces a quiescent energy phenotype in human macrophages, characterized by diminished flux through central carbon metabolism.^51^

We implemented a backtracking strategy to further elucidate how Mtb modulates host metabolism. We first identified host metabolic genes whose dysregulation could plausibly account for the observed shifts in metabolic fluxes. We then interrogated the host-pathogen interaction network to uncover potential functional links between these host genes and Mtb-secreted proteins. Through *in-silico* gene knockdown and overexpression analyses, we predicted 18 host metabolic genes whose collective perturbation accounts for the metabolic changes observed during infection. Nine of the 18 genes encode solute carriers and transporters (SLC25A1, SLC22A5, SLC25A15, SLCO2A1, MT-CO2, MT-CO3, MMAA, ABCD1, and ACOT8) involved in the trafficking of fatty acids, bile acids, amino acids, nucleotides, prostaglandins, and TCA cycle intermediates across mitochondrial and plasma membranes.^50^^,^^52^^,^^53^^,^^54^^,^^55^^,^^56^^,^^57^ Their enrichment among the top-ranked metabolic regulators reflects the critical role of membrane transport in host-pathogen metabolic crosstalk and is consistent with Mtb’s well-established dependence on host-derived nutrients for intracellular survival. Their emergence as central network nodes suggests that membrane transport regulation may represent an important component of host metabolic remodeling during infection. Further, pathway enrichment analysis indicated that these 18 genes are involved in key metabolic processes, including fatty acid, amino acid, bile acid, nucleotide, and vitamin metabolism. Several identified genes had established roles in tuberculosis (TB) pathogenesis. LTA4H, a gene involved in leukotriene metabolism, has been associated with TB susceptibility and the regulation of inflammatory responses.^58^ Upregulation of PKM is known to potentiate the HIF1*α*-mediated Warburg effect, promoting a shift toward glycolysis that supports an antimicrobial immune phenotype.^59^ Similarly, upregulation of FASN facilitates the formation of lipid-rich environments conducive to Mtb persisting within the host.^60^ Metabolic rewiring involving glycolytic remodeling and altered lipid metabolism has also been strongly associated with macrophage polarization dynamics during TB infection. Previous studies have demonstrated that Mtb can modulate macrophage polarization toward pathogen-favorable M2-like metabolic states characterized by enhanced lipid accumulation and altered immunometabolic signaling.^61^^,^^62^^,^^63^ Therefore, the identification of PKM- and FASN-associated metabolic perturbations through the proposed backtracing approach further supports its biological relevance for capturing infection-associated macrophage metabolic adaptations. Among the 18 identified host metabolic modulators, six were upregulated during Mtb infection. Selective pharmacological inhibitors were commercially available for three of these modulators—PKM, GOT1, and LTA4H—and inhibition of these host targets significantly reduced the intracellular survival of wild-type H37Rv, providing functional evidence that the identified host metabolic modulators represent promising candidates for HDT.

To link the expression or function of these 18 host metabolic genes with cosponsoring Mtb proteins, we analyzed available data on Mtb secretome proteins reported to interact with host transcription factors (TFs). Leveraging literature mining and protein-protein interaction (PPI) network analysis, we constructed a minimal directed host-pathogen interaction network linking 13 host metabolic gene products to 9 secreted Mtb proteins. This systems-level network provides a framework for generating mechanistic hypotheses regarding how Mtb may influence host metabolic pathways. To assess the functional relevance of these Mtb proteins, we generated CRISPRi-based knockdown (KD) strains of H37Rv for each candidate gene. Notably, KD strains for *mce3E*, *fadA2*, and *ptpA* exhibited significantly reduced survival in infected macrophages, while showing no growth defects under *in vitro* culture conditions. These findings suggest that while these genes are non-essential for intrinsic bacterial growth, they are conditionally essential within the host environment, consistent with roles in host adaptation that may include modulation of host metabolic processes. This is consistent with prior reports demonstrating similar *in vitro* and *ex vivo* growth kinetics for Mtb strains lacking *ptpA*,^10^ supporting the notion that these genes primarily function in host adaptation rather than core bacterial physiology. Network analysis further revealed that Mce3E, FadA2, and PtpA associate with six, four, and six host metabolic targets, respectively, supporting their potential involvement in host-pathogen metabolic crosstalk.

Mce3E is a mammalian cell entry (Mce) protein of Mtb that facilitates immune evasion by suppressing host cytokine expression through inhibition of the ERK1/2 signaling pathway.^41^ FadA2, an Mtb enzyme involved in fatty acid metabolism, has also modulated the host MAPK signaling pathway.^37^^,^^64^ Mtb tyrosine phosphatase (PtpA), is a secreted virulence factor that enters the cytosol of infected macrophages and disrupts key cellular pathways, including phagosome maturation, innate immune signaling, apoptosis, and lipid metabolism.^17^ Interestingly, targeted proteomics revealed that these three Mtb proteins modulate the expression of key host metabolic proteins. The secreted protein Mce3E altered the levels of ACAA2, AK3, MMAA, PKM, SLC25A1, and SLCO2A1. FadA2 modulated the expression of AK3 and SLCO2A1, while PtpA affected the expression of ACAA2 and MMAA genes. These host proteins are primarily associated with mitochondrial function and lipid metabolism, two key host pathways that Mtb modulates to survive within the host.^51^^,^^65^ Knockdown of the bacterial genes restored the expression of several mitochondrial proteins, including SLC25A1, MMAA, ACAA2, AK3 and PKM, supporting a role for these bacterial proteins in modulating host mitochondrial processes. This is further supported by evidence that PtpA dephosphorylates HADHA, an upstream regulator of ACAA2 in the mitochondrial fatty acid beta-oxidation pathway, thereby diminishing mitochondrial function.^17^^,^^66^ Importantly, pharmacological rescue of the intracellular growth defects of the *mce3E* and *ptpA* KD strains supports the predicted host-pathogen interactions and suggests that these bacterial proteins promote intracellular survival by manipulating specific host metabolic regulators. This provides functional evidence for a host-pathogen metabolic mechanism underlying Mtb persistence within macrophages.

Our study also suggests that the Mtb proteins Mce3E, FadA2, and PtpA significantly shape the host macrophage metabolome during infection. Untargeted metabolomic profiling of THP-1 macrophages infected with KD strains lacking each protein revealed widespread metabolic alterations, indicating that these proteins are associated with broad alterations in host metabolic pathways. Most of the metabolite changes observed in wild-type H37Rv-infected macrophages were reversed in cells infected with these KD strains, underscoring the involvement of these bacterial proteins in manipulating host metabolism. Notably, changes in phospholipids, bile acids, and fatty acids were prominent in H37Rv-infected cells. They were reversed in KD strains, particularly *ptpA* and *fadA2*, suggesting their involvement in remodeling the host lipids in ways that favor the survival of bacteria inside the cell. Pathway-level analysis further revealed that knockdown of these Mtb proteins partially restored metabolic signatures associated with pathways disrupted in wild-type infection. For example, the *mce3E* and *ptpA* KD strains showed enrichment in pathways associated with beta-oxidation of fatty acids, corresponding with upregulation of host enzymes such as ACAA2 and AK3. These findings are consistent with previous evidence that Mtb influences host fatty acid oxidation, and suggest that disruption of this modulation may compromise intracellular bacterial fitness.^67^^,^^68^^,^^69^^,^^70^

Interestingly, the mitochondrial citrate carrier SLC25A1, essential for prostaglandin E2 (PGE2) synthesis, was upregulated in macrophages infected with the *mce3E* KD strain, while SLCO2A1, which mediates PGE2 cytosolic transport, was elevated in macrophages infected with both *mce3E* and *fadA2* KD strains. SLC25A1 has been reported to contribute to the production of reactive oxygen species (ROS), nitric oxide (NO), and prostaglandins in host cells,^52^^,^^71^ all of which are critical components of the host innate immune response against Mtb. Further, Mtb has evolved multiple mechanisms to suppress these host defenses, including inhibition of ROS generation, evasion of ROS-mediated cytotoxicity,^72^ and active suppression of NO production, thereby dampening critical anti-mycobacterial responses within host macrophages.^73^ It has also been reported that Mtb impairs host immunity by suppressing PGE2 production, thereby shifting macrophage fate from apoptosis to necrosis and promoting bacterial escape and dissemination.^74^ Enhancing host PGE2 levels has also been proposed as an HDT to restrict Mtb replication.^75^^,^^76^ Here, we observed elevated PGE2 levels in macrophages infected with the *mce3E* and *fadA2* KD strains compared with wild-type, suggesting that these bacterial proteins may suppress prostaglandin-mediated immune responses. This observation was confirmed by our finding that exogenous PGE2 treatment significantly reduced the intracellular survival of wild-type H37Rv in infected macrophages.

While our study experimentally validated several predictions of the proposed reverse-mapping framework, many computationally inferred mechanisms remain to be explored. Among the 18 predicted host metabolic modulators, only three could be experimentally evaluated because selective pharmacological inhibitors were available. The remaining predicted host metabolic modulators represent promising candidates for future validation using pharmacological or genetic perturbation approaches. Similarly, our rescue experiments uncovered previously unrecognized host-pathogen metabolic mechanisms underlying Mtb persistence within macrophages. Additional mechanistic insights may be obtained by perturbing other predicted host metabolic regulators in the corresponding Mtb knockdown strains using selective activators or inhibitors. Furthermore, we demonstrated that exogenous PGE2 significantly reduced intracellular survival of wild-type H37Rv, supporting a protective role for prostaglandin-mediated host responses. Additionally, our findings suggest that the host proteins SLC25A1 and SLCO2A1 may also be involved in this interaction, implying a role for them in modulating Mtb growth within the host. Notably, reduced expression of SLC25A1 has been reported to limit the production of pro-inflammatory mediators in both primary human macrophages and U937 cells.^52^ SLC25A1, therefore, represents a potential HDT candidate that warrants further investigation.

### Limitations of the study

To our knowledge, this is the first study to combine genome-scale metabolic modeling with host-pathogen interaction analysis to infer candidate host metabolic modulators and their potential links to bacterial effectors. While our findings provide valuable insights, a key limitation is the lack of experimental validation using macrophages with altered expression of the identified host genes. An additional limitation stems from the assumptions inherent to constraint-based modeling: the framework assumes steady-state metabolism, which may not fully capture the dynamic, time-resolved shifts occurring during active infection, and flux predictions were derived from transcriptomic data as a proxy for metabolic activity rather than direct enzyme kinetics or fluxomics measurements. Additionally, while the genome-scale modeling approach employed here prioritizes network-wide coverage, it does not resolve specific metabolic bottlenecks, pathway control points, or the precise selective advantage these perturbations confer to Mtb, and a more granular mechanistic dissection of individual dysregulated pathways represents an important direction for future work. Future studies that validate these findings and identify key proteins as potential therapeutic targets for tuberculosis represent a promising direction for further research. Expanding this approach to construct a comprehensive interaction network encompassing all secreted Mtb proteins and their corresponding host targets could reveal additional modulators and broaden the repertoire of host-based targets for tuberculosis therapy. Although lung tissue transcriptomic datasets capture the global host response to infection, they may not fully reflect macrophage-specific metabolic programs. Since macrophages are the principal intracellular niche for Mtb, THP-1-derived macrophages were used for experimental validation as an established *in vitro* model of TB.^77^ This approach, which identifies candidate genes from *in vivo* lung tissue and validates them in macrophage models, is commonly used in TB research.^78^^,^^79^ However, this system does not fully recapitulate the metabolic and immunological complexity of primary macrophages; therefore, discrepancies between tissue-level and cell-type-specific responses remain a limitation of the current study. Although the experimental data at 48 hpi differ temporally from the 4-week *in vivo* model, this time point captures the late metabolic adaptation phase of Mtb-infected macrophages and is widely employed in metabolomics and flux analyses of THP-1 macrophages.^24^^,^^80^^,^^81^ Future validation in primary macrophages, including bone marrow-derived macrophages, as well as extended time-course experiments, would further strengthen the biological relevance and temporal scope of the proposed framework.

Despite current limitations, the insights gained from this study would help advance our understanding of tuberculosis pathogenesis and contribute to the development of effective HDTs against tuberculosis. While the individual computational and experimental approaches employed in this study have been previously described, their integration into a reverse-mapping framework that infers candidate Mtb effectors from host metabolic perturbations represents a distinct conceptual strategy. This host-to-pathogen inference workflow provides a systematic means of prioritizing bacterial factors associated with host metabolic reprogramming for subsequent experimental validation. We believe the conceptual framework presented here, integrating transcriptomics, metabolic constraint-based modeling, proteomics, metabolomics, and functional validation, can be broadly applied to other host-pathogen systems.

## Resource availability

### Lead contact

Requests for further information and resources should be directed to and will be fulfilled by the lead contact, Dr. Samrat Chatterjee (samrat.chatterjee@thsti.res.in).

### Materials availability

This study did not generate new materials.

### Data and code availability

• The targeted MRM data were available via ProteomeXchange with the identifier PXD067154, and the metabolomics data were available via Metabolomics Workbench under the study ID ST004099 [https://doi.org/10.21228/M8SV6X].
• The codes are available at https://github.com/samrat-lab/GSMM_tuberculosis.
• Any additional information required to reanalyze the data reported in this paper is available from the lead contact upon request.

## Acknowledgments

The authors acknowledge the BSL-3 facility at THSTI for all Mtb-related experiments. A.K.P acknowledges financial support from the Indian Council of Medical Research (ICMR), Government of India (grant no. EM/Dev/SG/212/7864/2023). The research of J.K is supported by the THSTI PhD Fellowship, and V.B is supported by the Council of Scientific and Industrial Research (India) (file no 09/1049(11494)/2021-EMR-I) PhD fellowship.

## Author contributions

J.K. and S.C. conceived and designed the study; J.K. and A.P. performed the computational analysis; V.B. performed biological experiments; A.B. and T.K.M. performed proteomics; P.M. and Y.K. performed metabolomics; J.K. complied the results; J.K., V.B., A.K.P., and S.C. interpreted the results; J.K., A.P., V.B., A.B., Y.K. wrote the original manuscript; T.K.M., A.K.P., and S.C. reviewed the manuscript; A.K.P. and S.C. supervised the work; all authors approved the final version.

## Declaration of interests

The authors declare no competing interests.

## STAR★Methods

### Key resources table

**Table:** 

REAGENT or RESOURCE	SOURCE	IDENTIFIER
**Bacterial and virus strains**		

*E. coli* XL1Blue	Dr. Amit Kumar Pandey Lab	N/A
Wild-type Mtb: *M. tuberculosis* H37Rv	Kind gift from Christopher M. Sassetti	N/A
Rv-WT Luciferase reporter: Rv:*lux*	Dr. Amit Kumar Pandey Lab	N/A
Rv-WT with control sgRNA (non-targeting) CRISPRi control: Rv:*lux ::ctrlsgRNA*_*KD*_	Dr. Amit Kumar Pandey Lab	N/A
Rv-WT lux reporter strain with *mce2E* gene knocked down using CRISPRi: Rv:*lux ::mce2E*_*KD*_	This paper	N/A
Rv-WT lux reporter strain with *mce3E* gene knocked down using CRISPRi: Rv:*lux ::mce3E*_*KD*_	This paper	N/A
Rv-WT lux reporter strain with *fadA2* gene knocked down using CRISPRi: Rv:*lux ::fadA2*_*KD*_	This paper	N/A
Rv-WT lux reporter strain with *pknG* gene knocked down using CRISPRi: Rv:*lux ::pknG*_*KD*_	This paper	N/A
Rv-WT lux reporter strain with *echA21* gene knocked down using CRISPRi: Rv:*lux ::echA21*_*KD*_	This paper	N/A
Rv-WT lux reporter strain with *Rv1463* gene knocked down using CRISPRi: Rv:*lux ::Rv1463*_*KD*_	This paper	N/A
Rv-WT lux reporter strain with *ptpA* gene knocked down using CRISPRi: Rv:*lux ::ptpA*_*KD*_	This paper	N/A
Rv-WT lux reporter strain with *gnd2* gene knocked down using CRISPRi: Rv:*lux ::gnd2*_*KD*_	This paper	N/A
Rv-WT lux reporter strain with *Rv3224* gene knocked down using CRISPRi: Rv:*lux ::Rv3224*_*KD*_	This paper	N/A

**Deposited data**		

Raw and analyzed targeted MRM data	N/A	ProteomeXchange with the identifier PXD067154
Raw and analyzed Metabolomics data	N/A	Metabolomics Workbench under the study ID ST004099

**Experimental models: Cell lines**		

THP-1 (male)	ATCC	ATCC® TIB-202; RRID:CVCL_0006

**Oligonucleotides**		

See Table S6 for list of primers used in the study	–	–

**Recombinant DNA**		

Zeo, Integrative, Lux expressing plasmid: pJW276	Kind gift from Prof. Sarah M. Fortune	N/A
Kan, attB integrative, target sgRNA and dCas9Sth expressing plasmid for transcriptional knockdown of target gene(s) in *M. tuberculosis*: pLJR965	Kind gift from Prof. Sarah M. Fortune	N/A
CRISPRi knockdown construct for targeting *mce2E* (*Rv0593*) in *M. tuberculosis*: pLJR965:*mce2E_sgRNA*	This paper	N/A
CRISPRi knockdown construct for targeting *mce3E* (*Rv1970*) in *M. tuberculosis*: pLJR965:*mce3E_sgRNA*	This paper	N/A
CRISPRi knockdown construct for targeting *fadA2* (*Rv0243*) in *M. tuberculosis*: pLJR965:*fadA2_sgRNA*	This paper	N/A
CRISPRi knockdown construct for targeting *pknG* (*Rv0410c*) in *M. tuberculosis*: pLJR965:*pknG_sgRNA*	This paper	N/A
CRISPRi knockdown construct for targeting *echA21* (*Rv3774*) in *M. tuberculosis*: pLJR965:*echA21_sgRNA*	This paper	N/A
CRISPRi knockdown construct for targeting *Rv1463* in *M. tuberculosis*: pLJR965:*Rv1463_sgRNA*	This paper	N/A
CRISPRi knockdown construct for targeting *ptpA* (*Rv2234*) in *M. tuberculosis*: pLJR965:*ptpA_sgRNA*	This paper	N/A
CRISPRi knockdown construct for targeting *gnd2* (*Rv1122*) in *M. tuberculosis*: pLJR965:*gnd2_sgRNA*	This paper	N/A
CRISPRi knockdown construct for targeting *Rv3224* in *M. tuberculosis*: pLJR965:*Rv3224_sgRNA*	This paper	N/A

### Experimental model and study participant details

#### Mtb strains and CRISPRi knockdown strains

Wild-type *Mycobacterium tuberculosis* H37Rv strain used in the study was a kind gift from Christopher M. Sassetti. CRISPRi-mediated knockdown strains targeting *mce2E*, *mce3E*, *fadA2*, *pknG*, *echA21*, *Rv1463*, *ptpA*, *gnd2*, and *Rv3224* were used in this study. The corresponding luciferase-expressing strains and CRISPRi constructs are listed in the key resources table. Knockdown expression was induced using anhydrotetracycline (ATc).

#### THP-1 macrophage model

THP-1 human monocytic cells (ATCC TIB-202; RRID:CVCL_0006) were used as the macrophage model for intracellular Mtb survival, growth rescue, targeted proteomics, and metabolomics experiments. THP-1 cells were differentiated into macrophage-like cells using phorbol 12-myristate 13-acetate (PMA) as described in the method details section. All infection and host-response experiments were performed using THP-1-derived macrophages.

#### Study participants

No human participants or human-derived clinical specimens were used in this study.

### Method details

#### RNA-seq. Data information and pre-processing

In this study, we used gene expression data (RNA-Seq.) from the H37Rv-infected lung tissue of mice.^79^ The dataset included three biological replicates of mice infected with H37Rv and two biological replicates of uninfected mice. The gene expression data provided had been previously quantile normalized.

#### Differential expression analysis of RNA-Seq. Data

We performed a two-sample *t* test to compare the transcriptomics profiles between these two groups. *p*-values were calculated using ‘ttest2’ function in MATLAB. Differentially expressed genes were defined as those with a fold change ≥1.2^82^^,^^83^ and *p*-value <0.05.

#### Reconstruction of condition-specific metabolic models

Condition-specific metabolic models were constructed using Mouse1,^26^ a generic genome-scale metabolic model of mouse metabolism. Mouse1 contains 13063 reactions, 2959 unique metabolic genes, and 8370 unique metabolites across nine cellular compartments (extracellular, peroxisome, mitochondria, cytosol, lysosome, endoplasmic reticulum, Golgi apparatus, nucleus, and inner mitochondria).

Transcriptomic data were organized into tissue-specific expression structures containing gene identifiers and corresponding expression levels. A transcript expression threshold of 0.5 was used during model extraction to distinguish lowly expressed genes from metabolically active genes. Reaction-associated expression values were subsequently estimated using the ‘mapExpressionToReactions’ function under the E-flux framework, where gene expression values were mapped onto metabolic reactions through logical GPR relationships. To obtain the condition-specific metabolic model, the task-driven integrative network inference for tissues (tINIT)^27^ was applied. We used the ‘getINITModel2’ function from the Reconstruction, Analysis and Visualization of Metabolic Networks (RAVEN) Toolbox 2.7.9^84^ of MATLAB to run the tINIT algorithm and set the expression threshold to 0.5. The algorithm consisted of 57 essential metabolic tasks^27^ as ‘taskStructure’ parameter, and mean gene expression values were used as the data input in ‘arrayData’ parameter. The parameter ‘removeGenes = true’ was used to remove lowly or non-expressed genes, while ‘useScoresForTasks = true’ enabled transcriptomic evidence to guide reaction retention during task optimization. Optimization runtime was controlled using ‘params.TimeLimit = 5000’ and while the remaining parameters were set to the default. Subsequently, the bounds of each reaction were fixed on these tINIT models by applying the E-flux algorithm.^28^ Finally, this process yielded metabolic models of lung tissue from H37Rv-infected and uninfected mice.

#### Flux based analysis

In general, metabolic models are under-determined systems with more unknowns than equations due to more reactions than metabolites, resulting in an infinite number of possible solutions. To address this, we performed flux variability analysis (FVA)^85^ on the generated model to determine the maximum and minimum feasible flux ranges for each reaction under the imposed constraints, thereby estimating the uncertainty associated with the predicted flux values.

To obtain the altered reactions, we compared the flux ranges *C* = [min *C* max *C*] and *D* = [min *D* max *D*] for each reaction obtained from FVA according the methods described in,^86^ where *C* and *D* represent the flux intervals for the uninfected and H37Rv-infected mice lung groups, respectively. The flux ranges are compared according to the following equations:

**Case I:** *C* and *D* belong to the positive coordinate

D>Cif((minC<minD)&(maxC≤maxD))|((minC≤minD)&(maxC<maxD)),

and

D<Cif((minD<minC)&(maxD≤maxC))|((minD≤minC)&(maxD<maxC)).

**Case II:** *C* and *D* belong to the negative coordinate

D<Cif((minC<minD)&(maxC≤maxD))|((minC≤minD)&(maxC<maxD)),

and

D>Cif((minD<minC)&(maxD≤maxC))|((minD≤minC)&(maxD<maxC)).

We also calculated the fold changes for each reaction using the mean of the corresponding flux ranges. Reactions with a fold change greater than 1.2,^82^^,^^83^ along with altered flux ranges determined by the previously defined equations, were considered significantly altered metabolic reactions.

#### *In-silico* metabolic network perturbation

To predict the effect of perturbations in the metabolic network, we used the robust Metabolic Transformation Algorithm (rMTA)^87^ available in Constrained-Based Reconstruction and Analysis (COBRA) Toolbox 3.0.^88^ The rMTA is a network-based perturbation approach designed to identify genes whose perturbation can drive a metabolic system from one physiological state toward another. This algorithm identifies a perturbation that drives the fluxes of altered reactions in the desired direction while keeping the fluxes of unchanged reactions as close as possible to their original values. For a target gene *g*, perturbations were implemented at the reaction level using gene-protein-reaction (GPR) associations. This analysis requires four inputs: (i) the flux distribution (*v*^*ref*^) of the source (here, uninfected mice lung) state, (ii) set of reactions whose flux value should be changed in the backward (*R*_*B*_) and forward (*R*_*F*_) direction to transform the source state into the target (here, H37Rv-infected mice lung) state, (iii) reconstructed condition-specific metabolic model of source state, and (iv) the desired network perturbation. We performed a uniform flux sampling technique, the gpSampler function deployed in the COBRA Toolbox 3.0.^88^ of MATLAB (using the Gurobi^TM^ Solver v9.5.2)^89^ and got 15738 (2 × No. of reactions) different flux solutions for each reaction in the source state. Next, we took the mean of each reaction’s flux solutions as the first input. Further, we classified the reactions in source state into the forward (*R*_*F*_), backward (*R*_*B*_) and unchanged classes (*R*_*S*_) in the following way: a reaction (*r*_*i*_) is in *R*_*F*_ if $v_{i}^{\mathrm{ref}}>0$ and it is up-regulated or $v_{i}^{\mathrm{ref}}<0$ and it is down-regulated. Similarly, a reaction (*r*_*i*_) is in *R*_*B*_ if $v_{i}^{\mathrm{ref}}>0$ and it is down-regulated or $v_{i}^{\mathrm{ref}}<0$ and it is up-regulated. The remaining reactions were unchanged (*R*_*S*_). This list was used as a second input. For the third input, the metabolic model of the uninfected mice’s lung tissue was used as the source state. We considered single-gene knockdown or overexpression as the fourth input for each metabolic gene. This analysis provides a robust transformation score (rTS),^87^ reflecting how much the network perturbation would transform the metabolic flux state from source to target. Full details of the algorithm can be found in Valcárcel, Luis V. et al.^87^

##### *In-silico* gene knockdown

We systematically performed gene knockdown (KD) simulations ranging from 25% to 100% with a step size of 25. This simulation was performed by reducing the upper and lower flux value range for all reactions associated with the target gene. Let *R*(*g*) denote the set of reactions associated with gene *g* obtained from GPR association. For KD simulations, the allowable flux range of each reaction *r* ∈ *R*(*g*) was reduced by a specified percentage relative to its original bounds. Specifically, if *LB*_*r*_ and *UB*_*r*_ denote the original lower and upper bounds of reaction *r*, then for a knockdown level *α* (*α* = 0.25, 0.50, 0.75, or 1.00), the modified bounds were:

$$LB_{r}^{KD}=\left(1-\alpha \right)LB_{r}$$

$$UB_{r}^{KD}=\left(1-\alpha \right)UB_{r}$$

The above procedure was then applied to calculate the rTS for each knockdown. The genes having rTS>0 for all KD conditions were selected for further analysis.

##### *In-silico* gene overexpression

We systematically performed single gene overexpression (OE) simulations ranging from 25% to 100% with a step size of 25. Gene OE was simulated by increasing the upper and lower flux ranges for all reactions associated with the target gene. Similarly to KD analysis, for OE simulations, the allowable flux range was expanded according to:

$$LB_{r}^{OE}=\left(1+\alpha \right)LB_{r}$$

$$UB_{r}^{OE}=\left(1+\alpha \right)UB_{r}$$

The impact of OE in transforming the metabolic state from source (uninfected mice lung) to target (H37Rv-infected mice lung) was predicted using rMTA, and the rTS was obtained as described previously. The genes having rTS>0 for every OE condition were selected for further analysis.

#### Capturing flux states after the metabolic network perturbations

Our goal was to capture the effects of these selected genes, identified through *in-silico* gene knockdown and overexpression analyses, on the source metabolic flux state. To achieve this, we simulated perturbations in the metabolic network by either knocking down the selected genes by 50% (i.e., 2-fold downregulation) or overexpressing them by 100% (i.e., 2-fold upregulation). The perturbed metabolic states were predicted for each case using rMTA, and the best-scenario flux profiles were taken as the perturbed flux state. Next, we compared the perturbed flux state with the source state to identify the reactions whose fluxes shifted from the source state to the target state (i.e., the uninfected mouse lung).

#### Selection of minimal subset from 147 selected perturbed genes set

We aimed to identify a smaller subset of genes (*G*_1_) from the set of 147 perturbed genes (*G*) whose perturbations (via gene knockdown or overexpression) could collectively account for nearly all observed metabolic alterations in the H37Rv-infected mouse model. To achieve this, we analyzed the perturbed metabolic reactions derived from rMTA analysis for each of the 147 gene-perturbed flux states, as well as for the wild-type (WT) H37Rv-infected mouse model.

##### Computation of the reaction coverage percentage

We assessed the percentage coverage of the total altered reactions observed in the WT H37Rv-infected mouse model for each gene perturbation using the following equation:

$$c_{k}=\frac{n\left(A_{k}\cap A_{\text{WT}}\right)}{n\left(A_{\text{WT}}\right)}\times 100$$

where,

• *k* = 1, …, 147: Gene perturbation indices
• *A*_*k*_: Set of altered reactions in the *k*^th^ gene-perturbed model
• *A*_WT_: Set of altered reactions in the WT model
• *n*(⋅): Cardinality (number of elements in the set).

##### Computation of the percentage of altered reactions in each metabolic subsystem

For each of the metabolic subsystems (*i*), we computed the percentage of altered reactions (*p*_*i*,*k*_) for the *k*^th^ gene-perturbed model using the following equation:

$$p_{i,k}=\frac{a_{i,k}}{t_{i}}\times 100$$

where,

• *k* = 1, …, 147: Gene perturbation indices
• *a*_*i*,*k*_: Number of altered reactions in subsystem *i* in the *k*^th^ gene-perturbed model
• *t*_*i*_: Total number of reactions in subsystem *i*.

Similarly, the percentage of altered reactions for the WT H37Rv-infected mouse model was calculated using the following equation:

$$p_{i,WT}=\frac{a_{i,WT}}{t_{i}}\times 100$$

where *a*_*i*,*WT*_ is the number of altered reactions observed in the WT model, and *t*_*i*_ is the total number of reactions in subsystem *i*.

##### Computation of the percentage of altered reactions in each metabolic subsystem

Here, the subsystem count ratio *R*_*k*_(*x*) represents how many metabolic subsystems are perturbed in a given *k*^th^ gene perturbation model, compared to a wild-type (WT) model, at a specific threshold of alteration *x%*. To calculate *R*_*k*_(*x*), we first quantified the number of metabolic subsystems in which the percentage of altered reactions exceeded a given threshold *x%*, where *x* = 50, 60, …, 100, for each gene perturbation condition as well as for the WT H37Rv-infected mouse model. This was done using an indicator function. Next, we compared the number of altered subsystems in the *k*^th^ gene perturbation model with that of the WT model to compute the subsystem count ratio (*R*_*k*_(*x*)), using the following equation:

$$R_{k}\left(x\right)=\frac{\overset{n}{\underset{i=1}{\sum }}I\left(p_{i,k}\geq x\right)}{\overset{n}{\underset{i=1}{\sum }}I\left(p_{i,WT}\geq x\right)}$$

where,

• *k* = 1, …, 147: Gene perturbation indices
• *x*: Threshold percentage, *x* ∈ {50, 60, …, 100}
• Indicator function, $I\left(\text{condition}\right)=\left\{\begin{array}{cc} 1 & \text{if}\;\text{condition}\;\text{holds}\\ 0 & \text{otherwise} \end{array}\right)$
• *p*_*i*,*k*_: Percentage of altered reactions in the *i*^th^ subsystem for the *k*^th^ gene-perturbed model
• *p*_*i*,*WT*_: Percentage of altered reactions in the *i*^th^ subsystem for the WT model
• *n*: Number of metabolic subsystems.

##### Gene ranking and final set determination

Gene perturbations were ranked for each alteration threshold (*x*) in descending order, primarily based on the subsystem count ratio (*R*_*k*_(*x*)) and secondarily on the reaction coverage (*c*_*k*_).

$$L\left(x\right)={\text{argsort}}_{k}\left(R_{k}\left(x\right),c_{k}\right)$$

where,

• *k* = 1, …, 147: Gene perturbation indices
• L(x): Ranked list of gene perturbations for threshold *x*
• argsort_*k*_(⋅): Sorting function in descending order, first by *R*_*k*_(*x*), then by *c*_*k*_.

From this ranking, we identified a subset of genes (*G*_1_) by selecting the intersection of the top 25 ranked genes across all percentage alteration thresholds (*x*), using the following equation:

$$G_{1}=\underset{x\in \left\{50,60,\ldots ,100\right\}}{⋂}G_{x}$$

Where,

• *G*_*x*_: Top 25 genes selected from L(x) at each threshold *x*
• *G*_1_: Genes that consistently appear in the top 25 across all thresholds

At the threshold *x* = 100%, only 25 of the 147 candidate genes exhibited a non-zero *R*_*k*_(100) value, defining a natural boundary imposed by the network topology. Of these 25 genes, those consistently ranked within the top 25 across all thresholds (*x* = 50%–100%) were retained as the final gene set. This cross-threshold intersection yielded 18 genes that exert robust, broad influence across multiple metabolic subsystems under both moderate and stringent definitions of pathway dysregulation, and these were selected for downstream analysis.

##### Hierarchical clustering

Hierarchical clustering was applied to both the rows and columns of the matrix representing the percentage coverage of altered reactions for each of the 147 gene perturbations across 125 subsystems, in order to obtain a reordered matrix. This was performed using the ‘Heatmap’ function from the ComplexHeatmap package in R.

#### Transcriptomic datasets and preprocessing used for validation

Transcriptomic datasets associated with Mtb H37Rv infection in mouse lung tissue were obtained from the Gene Expression Omnibus (GEO) repository for expression validation. GEO was queried using the keywords “mouse lung tissue” and “H37Rv infection”, resulting in the selection of two independent datasets: GSE137093^30^ and GSE23014.^31^ GSE137093 consisted of RNA-sequencing data generated from H37Rv-infected mouse lung tissue. Processed expression values deposited in GEO were used directly in this study. According to the original publication, raw sequencing reads were adapter-trimmed and filtered with Trimmomatic v0.36, aligned to the *Mus musculus* reference genome (Ensembl GRCm38 release 86) with HISAT2 v2.0.4, and quantified at the gene level with HTSeq v0.6.1. Raw count data were subsequently normalized using DESeq2 v1.12.4, and variance-stabilizing transformation (VST) was applied to generate normalized log_2_ expression values. The VST-normalized expression matrix deposited in GEO was used. GSE23014 consisted of microarray expression profiles obtained from H37Rv-infected mouse lung tissue. Processed log_2_ GC-RMA normalized expression values deposited in GEO were used directly. According to the original study, GC-RMA normalization was performed to correct for background noise and probe-specific effects and to generate normalized gene expression estimates. Expression matrices for both datasets were log2-transformed, and differential expression was quantified as the difference between mean log2 expression values across groups. A two-sample *t* test was performed to compare transcriptomic profiles between the two groups, with *p*-values calculated using the ‘ttest2’ function in MATLAB.

#### Identification of interconnection between host and Mtb proteins

We aimed to construct a host-pathogen interaction network to identify secreted or extracellular vesicle (EV) proteins of Mtb that may modulate the expression or function of key host metabolic genes,^90^^,^^91^^,^^92^ potentially driving pathogen-favoring metabolic perturbation. To do this, we performed transcriptional factor (TF) enrichment analysis for the selected host metabolic genes using Enrichr,^93^ querying three libraries: ENCODE and ChEA, TRANSFAC and JASPAR PWMs, and TRRUST.

We then constructed a host-pathogen interaction dataset through systematic manual literature curation. Initially, prioritized host metabolic proteins, and enriched TFs were individually queried against PubMed, Google Scholar, and published host-pathogen interaction databases like HPIDB 3.0,^94^ BioGRID^95^ to identify reported interactions with Mtb proteins. Interactions identified through literature mining were manually examined and retained only when supported by published studies. The corresponding Mtb proteins were additionally evaluated for evidence of secretion or EV association using published studies. For those TFs with no reported direct interactions, we retrieved their immediate upstream regulators from the SIGNOR v3.0.^96^ database. We then attempted to link these upstream regulators to Mtb proteins through the same above literature mining process, thereby inferring potential indirect interactions. The resulting interaction network therefore represents a manually curated framework integrating reported host-pathogen associations with Mtb-secreted or EV-associated proteins (see Table 1).

#### Bacterial strains and growth conditions

The wild-type *Mycobacterium tuberculosis* (Mtb) H37Rv strain served as the parental strain for all experiments in this study. Bioluminescent Mtb was generated by transforming the wild-type strain with an integrative plasmid encoding luciferase (pJW276).^97^ Bacteria were cultured in Middlebrook 7H9 liquid medium supplemented with 0.2% glycerol, 0.05% Tween 80, and 10% oleic acid-albumin-dextrose-sodium chloride (OADS) enrichment. Selective antibiotics kanamycin (30 *μ*g/mL) and zeocin (25 *μ*g/mL) were added as needed. Unless otherwise indicated, anhydrotetracycline (ATc) was used at a final concentration of 100 ng/mL. Cultures were incubated at 37°C with shaking at 150 rpm. For long-term storage, Mtb strains were stored as glycerol stocks at −80°C in a 25% glycerol solution.

#### Generation of Mtb CRISPR interference knockdown strain

To achieve transcriptional knockdown of Mtb genes using CRISPR interference (CRISPRi), the plasmid pLJR965 (Addgene plasmid #115163) was digested with BsmBI (New England Biolabs) and gel-purified. Target-specific single-guide RNA (sgRNA) sequences were generated by annealing complementary oligonucleotides and cloned into the digested plasmid. PCR confirmed the knockdown constructs and subsequently transformed them into the bioluminescent Mtb H37Rv strain.^98^

For gene knockdown, cultures were grown to mid-log phase, diluted to an optical density (OD600) of 0.0125 in fresh 7H9 medium, and treated with or without ATc at a final concentration of 100 ng/mL (Cayman Chemical) to maintain target gene knockdown. Growth was monitored by measuring culture luminescence using a multimode microplate reader (SpectraMax Pro ID3, Molecular Devices). Knockdown efficiency was further evaluated by quantifying target gene expression levels using quantitative real-time PCR (qRT-PCR).

#### RNA extraction and qRT-PCR

Total RNA was extracted from various Mtb knockdown strains, with or without ATc treatment (100 ng/mL), using TRIzol reagent (TaKaRa) following the manufacturer’s instructions. Briefly, bacterial cells were washed with PBS and resuspended in TRIzol. Cell lysis was performed by bead-beating with 0.22 *μ*m zirconia beads for five cycles, each followed by a 1-min cooling step. RNA was isolated using chloroform-isopropanol extraction and further purified through ethanol precipitation. Turbo DNase I (Ambion) was used to treat the resulting total RNA to remove genomic DNA contamination. A total of 3 *μ*g of DNase-treated RNA was used to synthesize complementary DNA (cDNA) using SuperScript IV Reverse Transcriptase (Invitrogen). Subsequently, 1 *μ*g of cDNA was utilized for quantitative real-time PCR (qRT-PCR) using SYBR Green reagents (TaKaRa). The housekeeping gene sigA was used as an internal control, and relative gene expression levels were calculated using the ΔΔCt method with respect to the specified control conditions.^99^

#### Macrophage infection

THP-1 monocytic cells were cultured in RPMI medium (Capricorn Scientific) supplemented with 10% fetal bovine serum (FBS; Invitrogen). Cells were seeded at a density of 5 × 10^4^ cells per well in a 96-well white plate (SPL) and differentiated into macrophage-like cells by treatment with 50 ng/mL phorbol 12-myristate 13-acetate (PMA; Sigma) for 36 h, followed by a 24-h rest period.

Single-cell suspensions of Mtb strains were prepared using the soft-spin method as described in^97^^,^^100^ and used to infect PMA-differentiated THP-1 cells at a multiplicity of infection (MOI) of 2. After a 4-h post-infection, extracellular bacteria were removed by washing the cells three times with pre-warmed PBS (Capricorn Scientific). Infected cells were maintained in an incubator at 37°C with 5% CO2. A complete RPMI medium containing 300 ng/mL ATc was added to the respective wells to induce the knockdown phenotype. At the indicated time points, luminescence was measured to assess the growth or survival of the knockdown strains relative to their wild-type strain.

For intracellular survival assays, THP-1 macrophages were infected with Mtb at an MOI of 10. On Day 1 post-infection, the inhibitors and PGE2 were added at various concentrations (10, 25, and 50 *μ*M). Gefitinib (25 *μ*M) was used as a positive control, and 0.05% DMSO served as the vehicle control. Intracellular Mtb luminescence was monitored until Day 4 post-infection. Each experimental group included six independent biological replicates. Data are presented as mean ± SD from independent biological replicates. Statistical significance was determined using ordinary one-way ANOVA followed by Dunnett’s multiple-comparison test for multiple-group comparisons, and paired or unpaired *t* tests where appropriate. For growth rescue assays, THP-1 macrophages were infected with Mtb at an MOI of 2. On Day 2 post-infection, the inhibitors were added at a final concentration of 25 *μ*M. During the experiment, anhydrotetracycline (ATc; 300 ng/mL) was used to induce gene knockdown, and 0.05% DMSO served as the vehicle control. Intracellular Mtb luminescence was monitored until Day 5 following inhibitor treatment. Each experimental group included five independent biological replicates. Data are presented as mean ± SD from independent biological replicates. Statistical significance was determined using ordinary one-way ANOVA followed by Dunnett’s multiple-comparison test for multiple-group comparisons, and paired or unpaired *t* tests where appropriate.

#### Targeted proteomics profiling

##### Sample preparation

The total protein was prepared by seeding THP-1 cells at a density of 2 × 10^6^ cells per well in 6-well plates and matured with 50 ng/mL of PMA for 36 h. Following differentiation, cells were infected with Mtb strains at an MOI of 5, and after the removal of extracellular bacteria, complete RPMI media was added, supplemented with 300 ng/mL of ATc to achieve the knockdown. Uninfected cells were maintained in parallel as controls. At 48 h post-infection, cells were washed with pre-warmed PBS, and 1 mL of ice-cold RIPA lysis buffer (Invitrogen) containing EDTA-free complete protease inhibitors (Roche) was added. The lysates were collected into pre-cooled microcentrifuge tubes (MCTs), vortexed for 15 min to lyse bacterial and host cells, and centrifuged at 12000 rpm for 10 min at 4°C. The supernatant was transferred to fresh pre-cooled MCTs, and chilled acetone was added to the lysate. The precipitated proteins were collected by centrifugation and redissolved in 8M Urea. Next, the total recovered protein from the acetone precipitation step is quantified using bicinchoninic acid (BCA) protein assay kit (Thermo Scientific, Cat # 23225). 100 *μ*g protein was taken from each sample for further reduction with 10 mM dithiothreitol at 56°C for 45 min, followed by alkylation with 20 mM Iodoacetamide at RT for 1 h in the dark. The reduced and alkylated proteins were digested with Pierce trypsin (Thermo Scientific, Cat # 90057) at a 1:20 ratio overnight.

##### Measurement of targeted peptides

Digested samples were held at 4°C in an autosampler until analysis via 10 *μ*L injections (typically 20 *μ*g total protein digest on-column). The autosampler loaded the sample onto a Sciex ExionLC Ultra high-performance liquid chromatography system fitted with a WATERS ACCUITY UPLC CSH C18 column (2.1 × 150 mm, 1.8 *μ*m) at 50°C with a flow rate of 0.2 mL/min. Elution was performed over a 70 min gradient consisting of 0%–2% B at 3 min, to 45% B at 57 min, to 95% B at 61 min, followed by a wash at 95% B for 4 min, and then returning to 2% B at 66 min for a 4 min equilibration (mobile phase A: H2O, 0.1% aqueous FA, mobile phase B: ACN, 0.1% aqueous FA). MRM-MS analyses were performed on Sciex QTOF 6500+ triple quadrupole mass spectrometer (AbSciex). Mass spectra were acquired in positive ion mode (ESI capillary ion spray voltage-5500 V; source gas temperature- 550°C; GS1-55, GS2-55 and Curtain Gas-32). The scheduled MRM method used a cycle time of 1 s and a 150 s detection window (declustering potential-100 V, Entrance potential-10 V and collision cell exit potential-15 V). The monitored MRM-MS transitions (at least 5 per peptide and 1 peptide per protein) together with their optimized collision energies are shown in Table S7.

##### Data processing

The acquired raw.wiff files from collected scheduled-MRM data were analyzed using the Analytics module within the Sciex OS platform (version 3.1.6.44, Sciex, MA, USA). Peaks were first extracted based on their alignment with expected retention times, then preprocessed using a noise-filtering algorithm and medium smoothing to reduce noise and enhance signal clarity. The following Specific quality criteria: 1) a minimum intensity threshold of 100 counts per second (cps), 2) signal to noise ratio (S/N) of more than or equal to 2, and 3) interference resolution of 50% were used to filter the peaks to ensure the selection of reliable and well-defined signals. Further, the selected peaks were manually reviewed to ensure an accurate detection and integration of the analyzed data for area under the curve (AUC) extraction of the chosen product ions, which will be used for downstream analysis. The targeted MRM data were available via ProteomeXchange with identifier PXD067154.

##### Differential expression analysis of targeted proteomics data

Protein abundance, as measured by AUC from targeted proteomics, was represented by the corresponding peptide abundances (Table S8). Each of the three H37Rv knockdown strains included three biological replicates under the -ATc (wild-type) condition and four under the +ATc (knockdown) condition, along with three biological replicates of uninfected samples. The data were sum-normalized, log10-transformed, and Pareto-scaled. To compare peptide peak intensities between groups, we used Student’s unpaired *t* test via the ‘ttest2’ function in MATLAB.

#### Metabolomics profiling

##### Sample preparation

THP-1 cells (2 × 10^6^) were seeded in 6-well plates and treated with 50 ng/mL PMA for 36 h to induce differentiation. After PMA treatment, the medium was replaced with fresh RPMI, and the cells were allowed to mature for an additional 24 h. Mtb infection was performed at a MOI of 5 and un-infected cells were maintained in parallel culture as a control. After 4 h of infection, extracellular bacteria were removed by three washes with pre-warmed PBS. A complete RPMI medium supplemented with 300 ng/mL ATc was added to the wells to induce knockdown. The infected cells were incubated for 48 h before being washed again with pre-warmed PBS. To quench metabolism and extract metabolites, 1 mL of chilled methanol (HPLC grade; Sigma) was added to the cells. The cells were harvested on ice using a scraper, and the resulting extracts were transferred to pre-cooled microcentrifuge tubes (MCTs) maintained at 4°C. The extracts were vortexed for 15 min and centrifuged at 12000 rpm for 10 min at 4°C. After centrifugation, the supernatants were transferred to fresh precooled MCTs (4°C), dried under vacuum and stored at −80°C until further analysis. The reconstituted samples (50% water: methanol (1v:1v)) were further analyzed by liquid chromatography-mass spectrometry (LCMS).^100^ A quality control (QC) sample was prepared to ensure instrument performance by pooling equal volumes from all individual samples.

##### Measurement of metabolites

All LC/MS-acquired data have been processed using the default setting of Progenesis QI for metabolomics (Water Corporation) software. The untargeted default settings workflow in Progenesis QI was used to perform retention-time alignment, feature detection, deconvolution, and elemental composition prediction. The Metascope plug-in in Progenesis QI has been used for the in-house library, with accurate mass, fragmentation pattern, and retention time for database search. We also used an online spectral library to identify metabolites. The identification was confirmed with the online spectral library. The cut-off for retention-time match was 0.15 min, and spectral similarity was greater than 30% fragmentation match in Progenesis QI. Peaks that had a coefficient of variation (CV) less than 30% in pool QC samples were kept for further analysis of data. Additionally, each detected feature was manually verified to select the right peaks.

##### Data processing

All LC/MS-acquired data have been processed using the default setting of Progenesis QI for metabolomics (Water Corporation) software. The untargeted workflow in Progenesis QI was used to perform retention-time alignment, feature detection, deconvolution, and elemental composition prediction. Metascope plug of Progenesis QI has been used for the in-house library with accurate mass, fragmentation pattern and retention time for database search. The identification was confirmed with the online spectral library. The cut-off for retention time match was 0.5 min, and spectral similarity was more than 30% fragmentation match in Progenesis QI. Peaks with a coefficient of variation (CV) less than 30% in pool QC samples were retained for further data analysis. Additionally, each detected feature was manually verified to select the right peaks.

##### Differential expression analysis of metabolomics data

Each of the three H37Rv knockdown strains with the -ATc (wild-type) condition has six biological replicates. All wild-type replicates were pooled for each metabolite, and outliers were identified using the quartiles method via the MATLAB function ‘isoutlier’. Wild-type samples containing outliers in fewer than ten metabolites were selected for further analysis. In this final matrix, we replace the remaining outliers with the mean value of the corresponding metabolite and got nine biological replicates for wild-type condition. Each of the three H37Rv knockdown strains included six biological replicates under the +ATc (knockdown) condition, along with six biological replicates of uninfected samples. The data were normalized by sum, log10-transformed, and scaled using the Pareto-scaling feature. Altered metabolites were selected based on a fold change cutoff of 1.2 and a *p*-value <0.05.^101^^,^^102^ We performed Student’s unpaired *t* test to compare the peak intensities of metabolites between the two groups using the ‘ttest2’ function in MATLAB.

##### Reporter pathway analysis

To identify significantly altered pathways, we conducted reporter pathway analysis.^103^ The *p*-values (p_metabolite_) obtained from the *t* test for each metabolite were transformed into Z-scores (Z_metabolite_) using the inverse normal cumulative distribution function (CDF). Pathway Z-scores (Z_pathway_) were then calculated by aggregating the Z-scores of the metabolites involved in each pathway, following the specified relation^103^

$${\text{Z}}_{\text{pathway}}=\frac{1}{\sqrt{r}}\sum_{\text{metabolite}=1}^{r}{\text{Z}}_{\text{metabolite}},r=\text{No.}\;\text{of}\;\text{metabolites}$$

Subsequently, the pathway Z-scores were converted back into *p*-values using the CDF. Pathways with *p*-values below 0.05 were identified as significant reporter pathways.

### Quantification and statistical analysis

All statistical analyses were performed using MATLAB unless otherwise stated. Data are presented as mean ± SD from independent biological replicates. For transcriptomic analysis, differences between three biological replicates H37Rv-infected and two biological replicates uninfected mouse lung samples were assessed using a two-sample *t* test implemented with the MATLAB ‘ttest2’ function. Differentially expressed genes were defined using a fold-change cutoff of 1.2 and a *p*-value <0.05.

For *in vitro* growth kinetics of Mtb CRISPRi knockdown strains, each experimental group included three biological replicates. Data are presented as mean ± SD from independent biological replicates. Data were analyzed using two-way ANOVA with Šídák’s multiple-comparisons test. Statistical significance was defined as ∗*p* = 0.0102, ∗∗*p* = 0.0043, ∗∗∗*p* = 0.0005, ∗∗∗∗*p* < 0.0001, and ns, P>0.9999.

For *ex vivo* intracellular growth kinetics, THP-1 monocyte-derived macrophages were infected with Mtb knockdown strains, and relative bacterial growth was quantified by luminescence. Each experimental group included five biological replicates. Data are presented as mean ± SEM, with individual biological replicates shown as points. Statistical significance was assessed against the −ATc group using ordinary one-way ANOVA with Holm-Šídák’s multiple-comparisons test. ∗, *p* < 0.01; ∗∗∗∗, *p* < 0.0001; ns, not significant.

For intracellular survival assays involving pharmacological perturbation, each experimental group included six biological replicates. Data are presented as mean ± SD from independent biological replicates. Statistical significance was determined using ordinary one-way ANOVAs with Dunnett’s multiple-comparison test, ratio paired *t* tests, or unpaired *t* tests. ∗*p* < 0.05, ∗∗*p* < 0.01, ∗∗∗*p* < 0.001, ∗∗∗∗*p* < 0.0001; ns, not significant.

For growth rescue assays, each experimental group included five biological replicates. Data are presented as mean ± SD from independent biological replicates. Statistical significance was determined using ordinary one-way ANOVAs with Dunnett’s multiple-comparison test, ratio paired *t* tests, or unpaired *t* tests. ∗*p* < 0.05, ∗∗*p* < 0.01, ∗∗∗*p* < 0.001, ∗∗∗∗*p* < 0.0001; ns, not significant.

For targeted proteomics and metabolomics analyses, differences in peptide or metabolite abundances between experimental groups were assessed using Student’s unpaired *t* test implemented with the MATLAB ‘ttest2’ function. Metabolites were considered altered using a fold-change cutoff of 1.2 and a *p*-value <0.05. Reporter pathway significance was determined using pathway Z-scores and a corresponding *p*-value <0.05. The data-processing procedures for targeted proteomics and metabolomics are described in the corresponding method details subsections. In the targeted proteomics analysis, each H37Rv knockdown strain included three biological replicates under the wild-type condition, four under the knockdown condition, and three uninfected controls. For metabolomics, each knockdown strain included six biological replicates under the knockdown condition, with six uninfected samples and the indicated wild-type replicates.

## Supplemental information

Document S1. Figures S1–S7

Table S1. Up- and down-regulated genes in Mtb-infected mouse lung tissue compared to uninfected

Table S2. Up- and down-regulated reactions in Mtb-infected mouse lung tissue model compared to uninfected model

Table S3. Differentially expressed genes with positive robust transformation score

Table S4. Percentage coverage of altered metabolic reactions for each of the 125 metabolic subsystems covered by the gene sets G, G1, and G − G1

Table S5. Transcription factors that regulate the expression of 18 selected host metabolic genes

Table S6. List of primers used in the study

Table S7. The monitored MRM-MS transitions together with their optimized collision energies

Table S8. Protein abundance derived from corresponding targeted peptide abundances
