# Supplementary material for: Metabolic modeling with reverse mapping links host perturbations to Mycobacterium tuberculosis
Source: iScience. 2026 Sep 17;29(10):117477. doi: 10.1016/j.isci.2026.117477 (PMC13602250; doi:10.1016/j.isci.2026.117477)
Supplement: Supplementary file 1 [file mmc1.pdf]

iScience, Volume *29*

Supplemental information

Metabolic modeling with reverse mapping links host perturbations to *Mycobacterium tuberculosis*

Jayendrajyoti Kundu, Vishawjeet Barik, Abhijit Paul, Ankit Biswas, Pallavi Mudgal, Yashwant Kumar, Tushar Kanti Maiti, Amit Kumar Pandey, and Samrat Chatterjee

Supplementary Figures

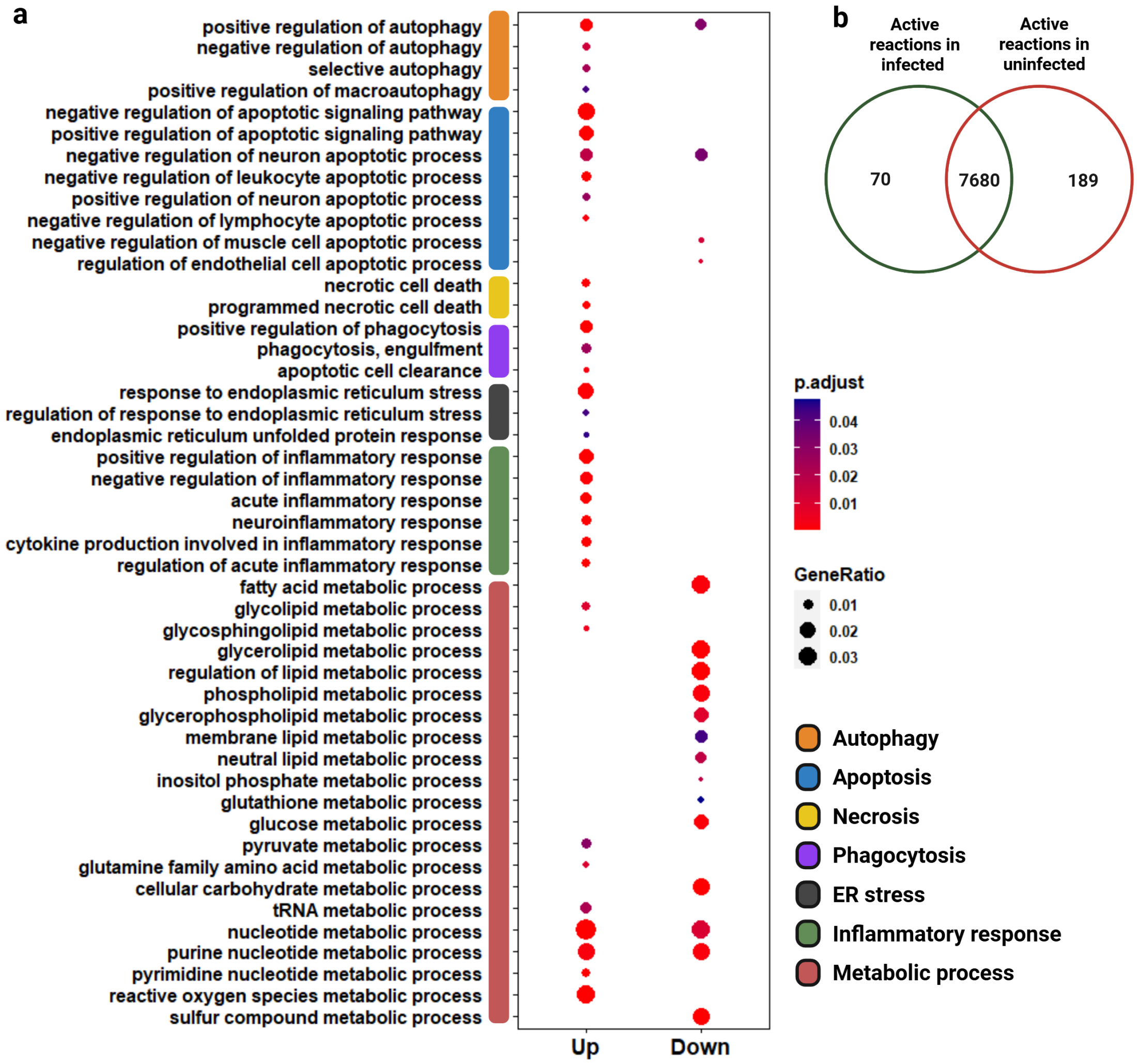

Figure S1: Pathway categories enriched by differential expressed genes (DEGs). a Gene ontology (GO) biological process over-representation test for DEGs. Benjamini-Hochberg correction was used with a q-value cut-off of 0.05, represented by a dot colour. Dot size measures how many DEGs are enriched in a certain biological process relative to the size of the total gene set. **b** Venn diagram for active reactions in H37Rv-infected and uninfected mice lung metabolic models.

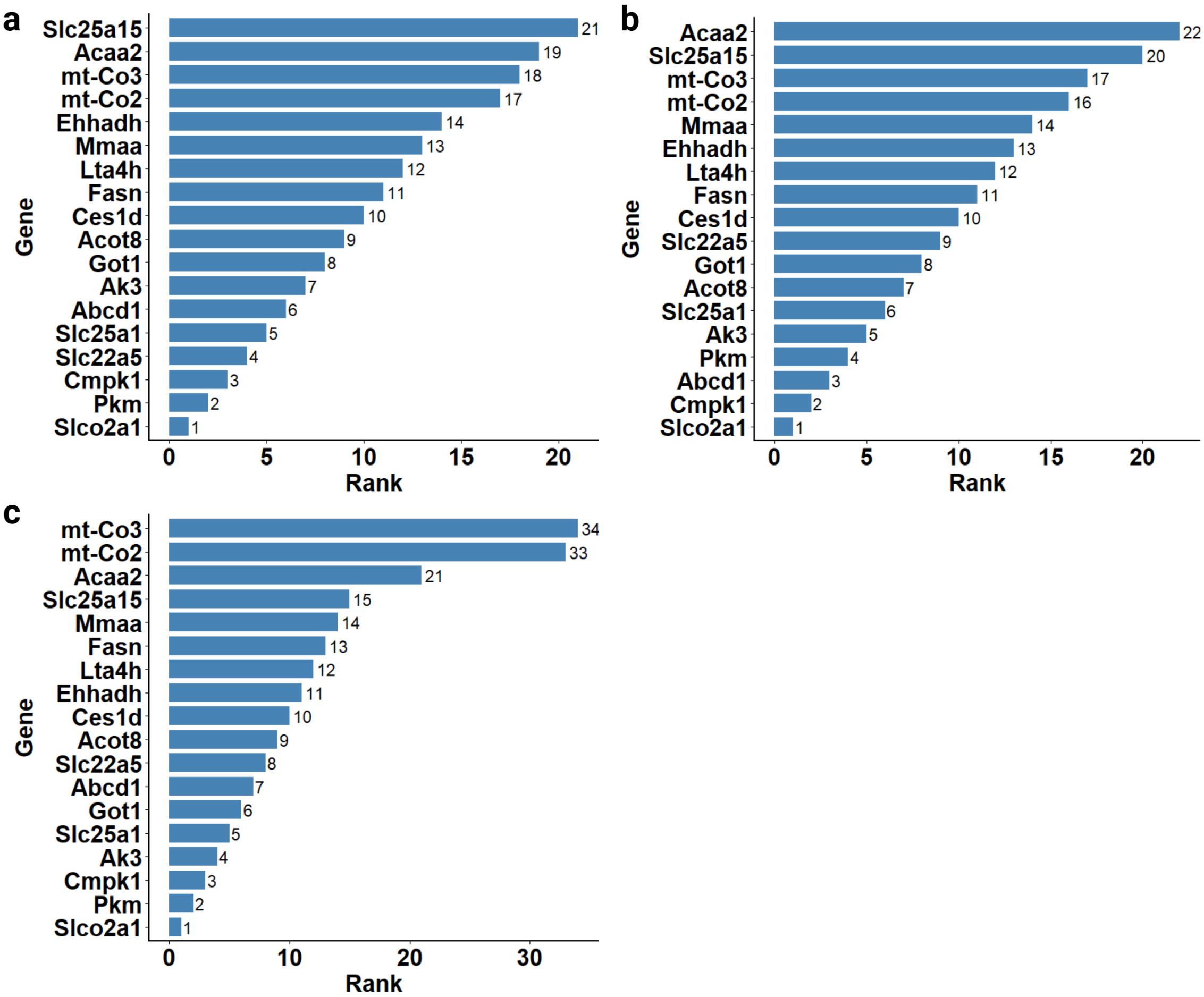

Figure S2: Ranking of gene set G_1 across different features. **a** Rank of G_1 within G based on clustering analysis. **b** Rank of G_1 within G based on altered reaction coverage. **c** Rank of G_1 within G based on the number of pathways achieving maximal percentage coverage.

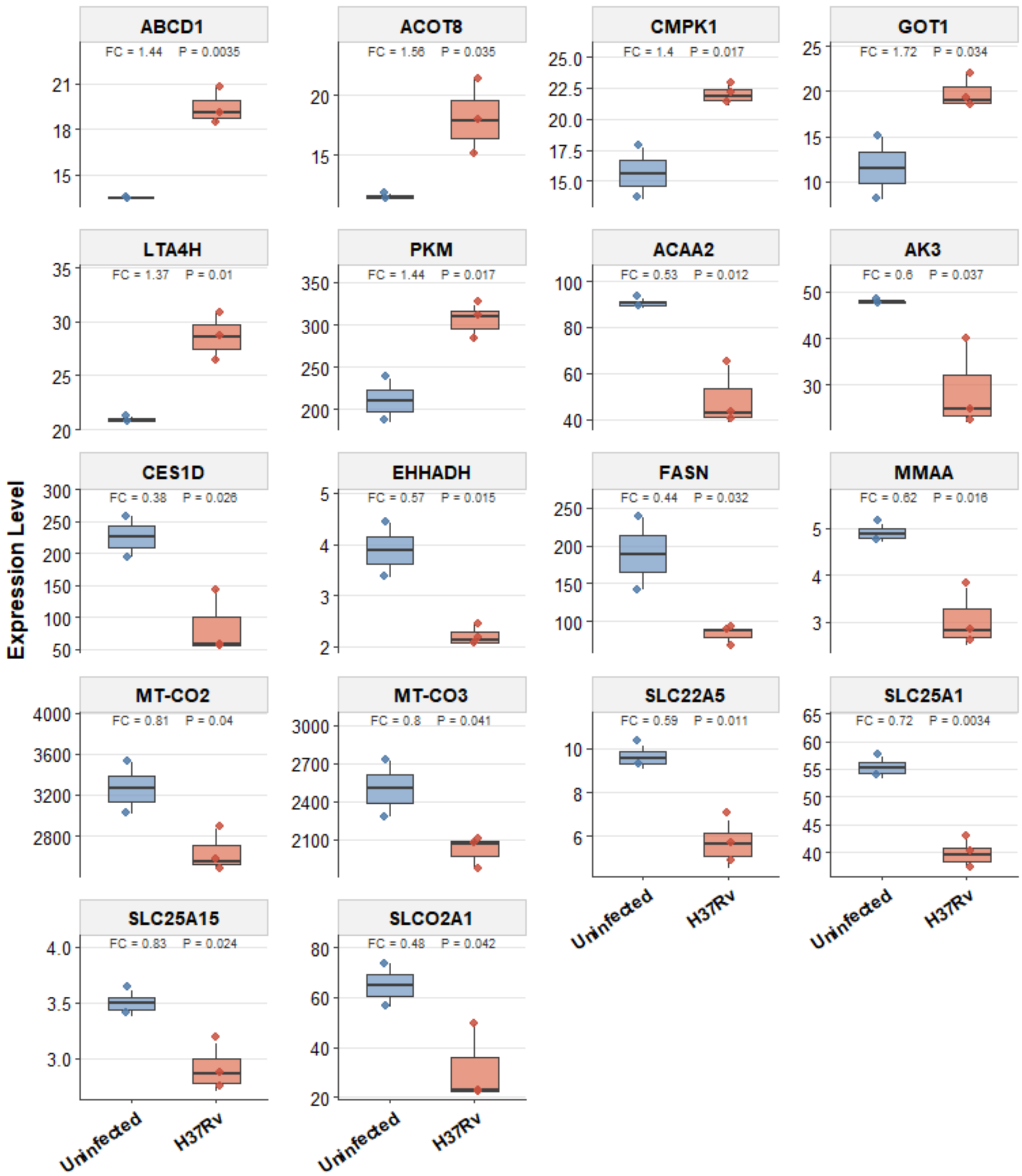

Figure S3: Expression profiles of the 18 prioritized host metabolic genes in Mtb-infected versus uninfected mouse lung samples. Box plots display expression levels across biological replicates. Fold-change values and corresponding p-values are shown within each plot. Statistical significance was assessed using Student's unpaired t-test.

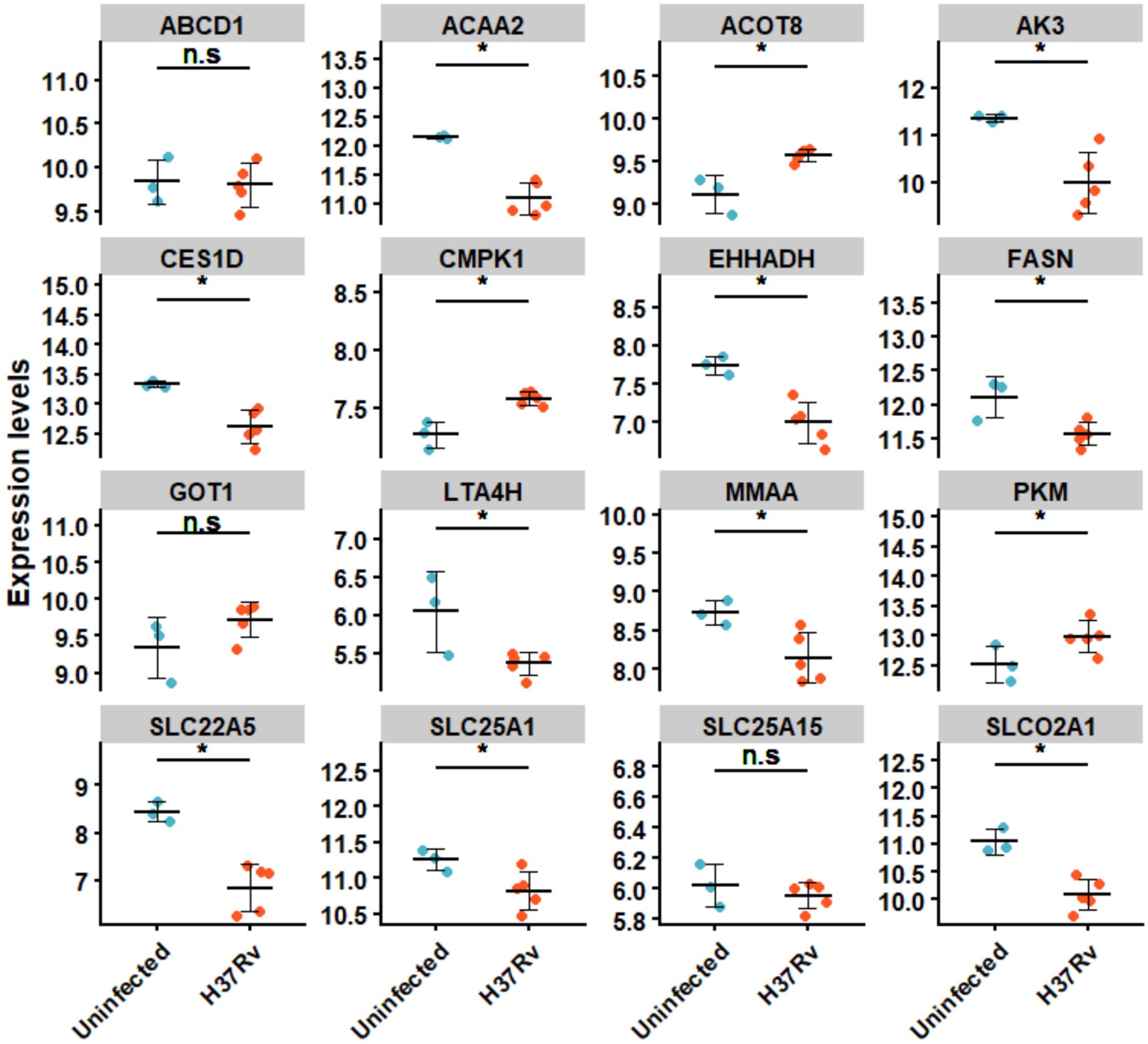

Figure S4: Expression patterns of the selected 18 genes in dataset GSE137093. Data are represented as mean ± SEM, with individual replicates indicated by data points. Statistical analysis was performed using Student's unpaired t-test between two groups, where *, P<0.05; n.s, non-significant.

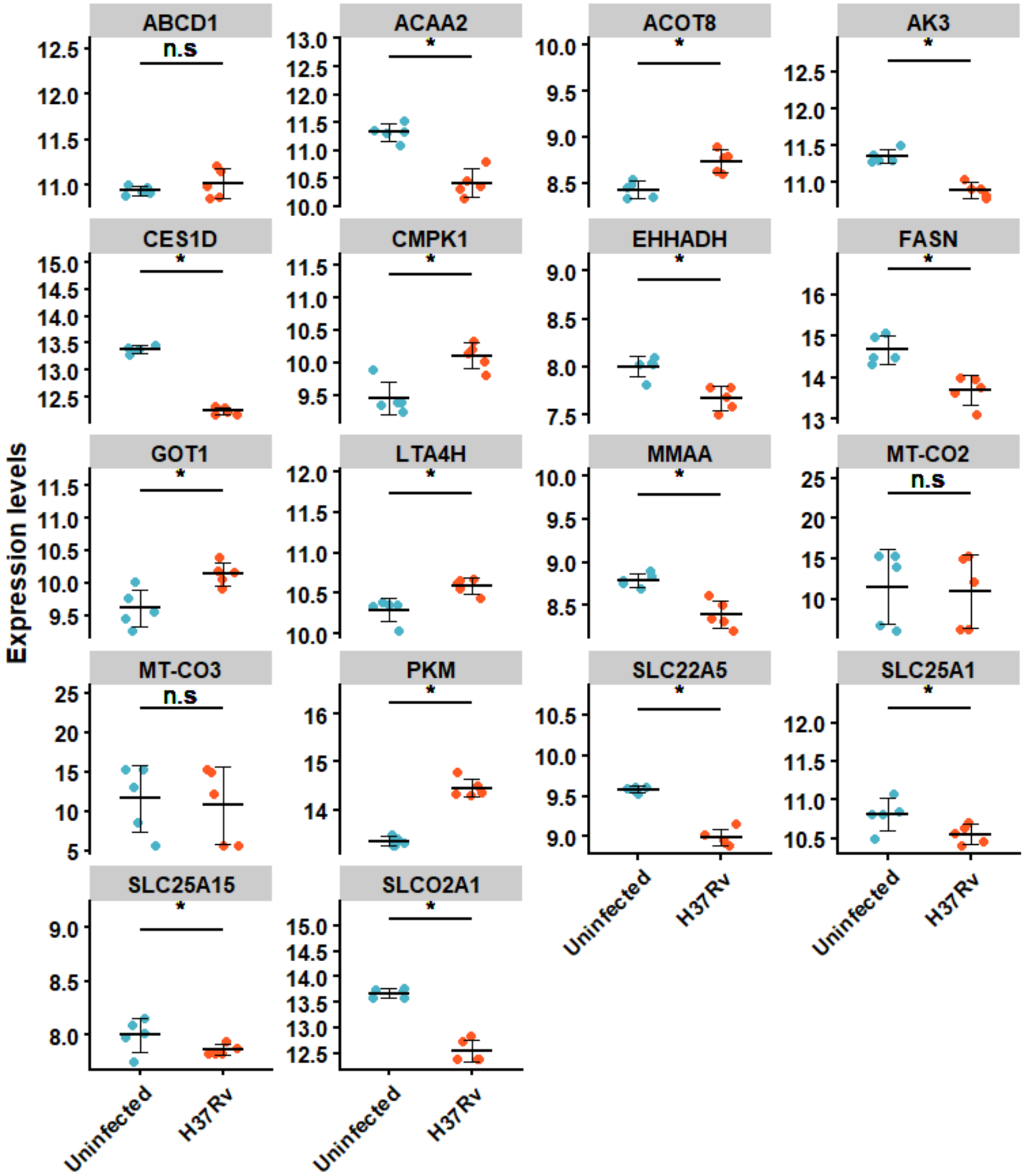

Figure S5: Expression patterns of the selected 18 genes in dataset GSE23014. Data are represented as mean ± SEM, with individual replicates indicated by data points. Statistical analysis was performed using Student's unpaired t-test between two groups, where *, P<0.05; n.s, non-significant.

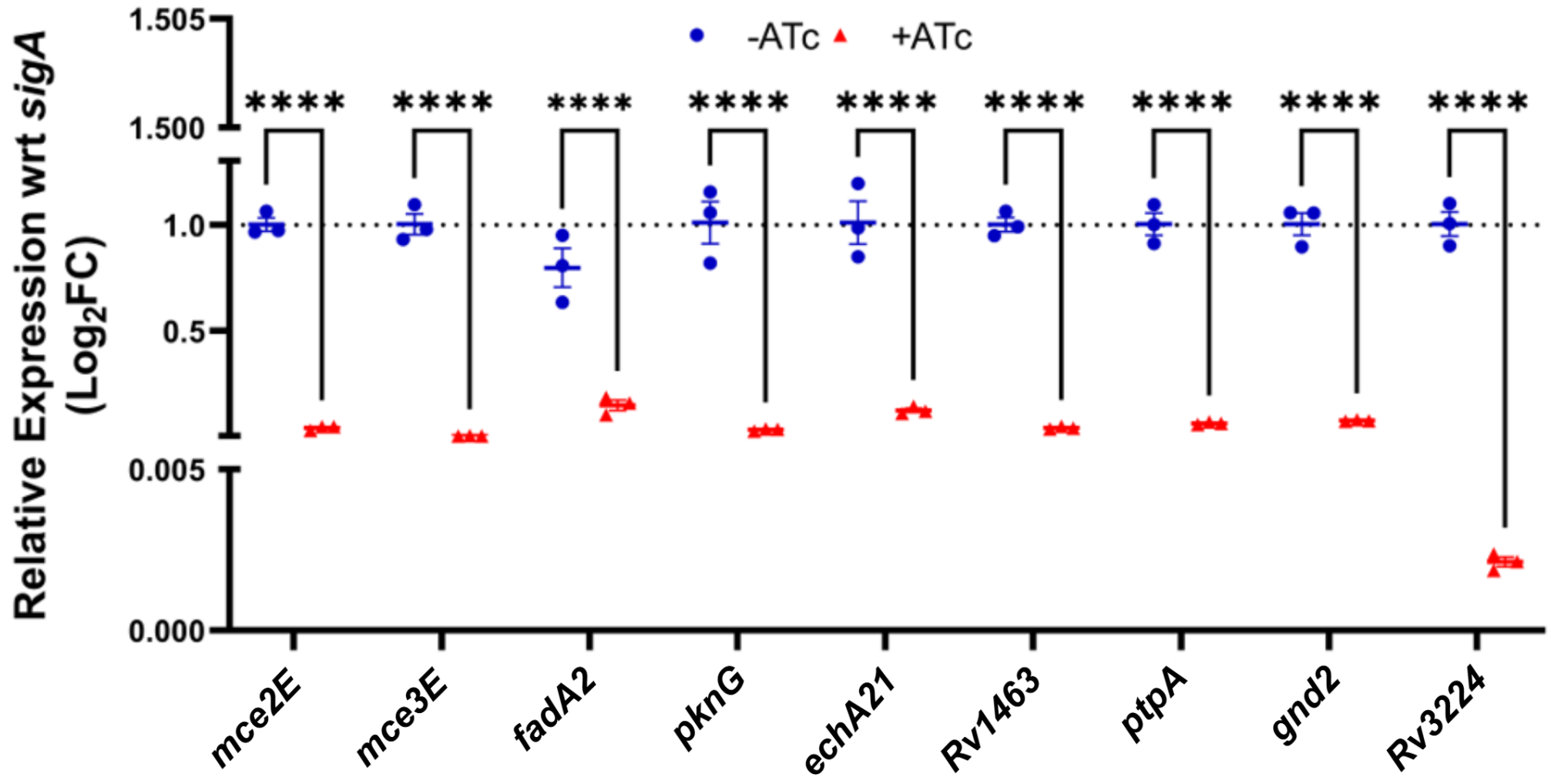

Figure S6: CRISPRi-mediated transcriptional knockdown of target genes in Mtb. Relative expression of Mtb gene knockdown strains using qRT-PCR analysis to confirm transcript depletion. Upon ATc (100ng/mL) treatment, individual genes' relative transcript levels were diminished and normalized with sigA. Data is represented as the mean ± SEM of the replicates indicated by points in the respective graphs. Comparisons for statistical significance have been made -ATc using two-way ANOVA (Šídák's multiple comparisons test). ****P<0.0001; ns, non-significant.

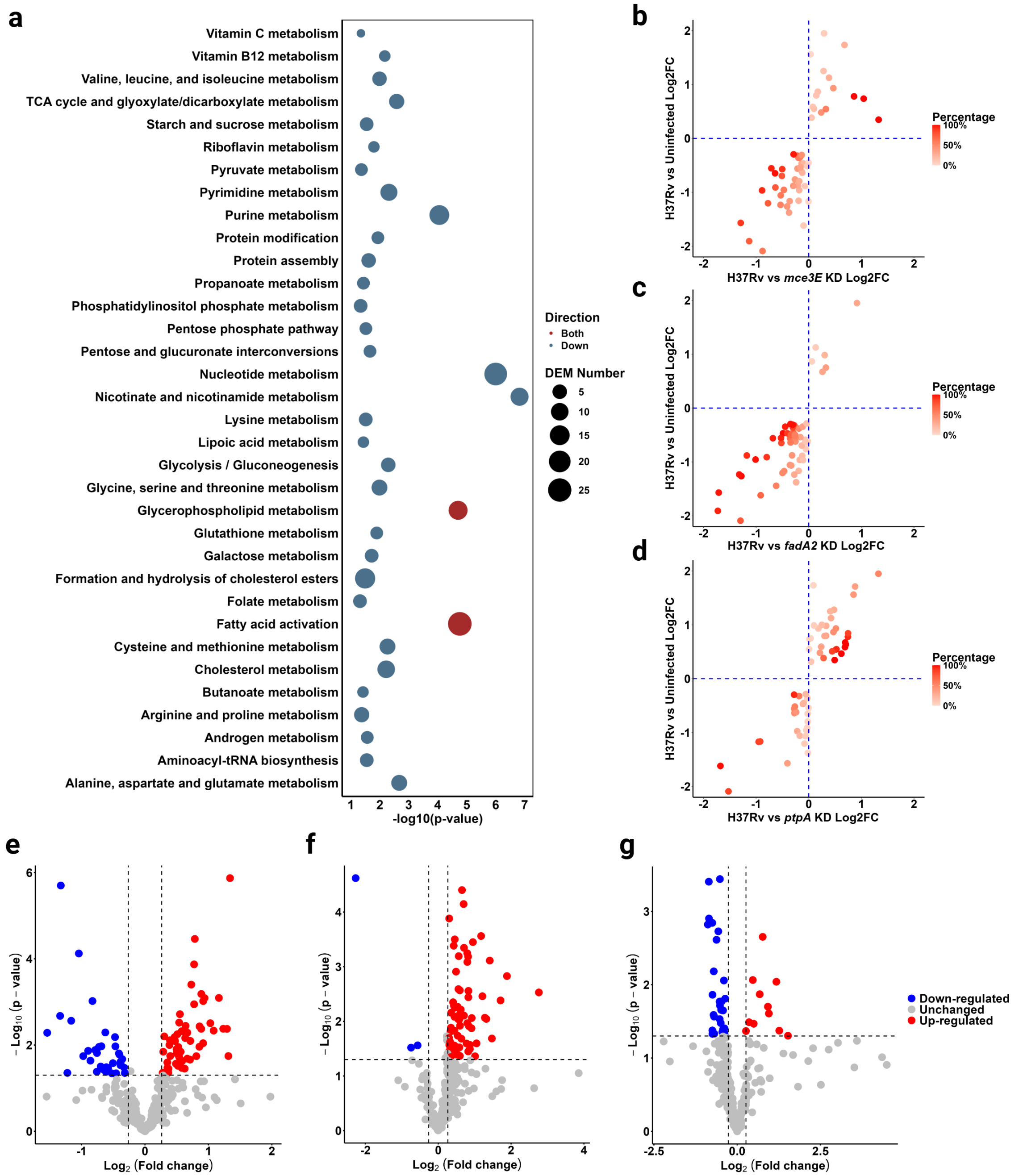

Figure S7: a Reporter pathway analysis was conducted to identify significantly altered pathways in macrophages infected with wild-type H37Rv strains compared to uninfected, with a significance threshold set at a p-value < 0.05. **b, c, d** Scatter plot of strength of reversing metabolite expression in *mce3E*, *fadA2*, and *ptpA* KD strains infected THP-1. **e, f, g** Volcano plot representation of differential expressed metabolites of *mce3E*, *fadA2*, and *ptpA* KD strains infected macrophage. Significant metabolites were selected using the fold change cut-off value of 1.2 and p-value cut-off of 0.05. The up-and down-regulated metabolites are highlighted here in red and blue colour, respectively.
