# Supplementary material for: Metabolic modeling with reverse mapping links host perturbations to Mycobacterium tuberculosis
Source: iScience. 2026 Sep 17;29(10):117477. doi: 10.1016/j.isci.2026.117477 (PMC13602250; doi:10.1016/j.isci.2026.117477)
Supplement: Supplementary file 2 [file mmc7.pdf]

REAGENT or RESOURCE	SOURCE	IDENTIFIER
Oligonucleotides		
mce2E-CR-F primer: GGGAGGGGCCAGCAAGACCGCCAG	Sigma-Aldrich	N/A
mce2E-CR-R primer: AAACCTGGCGGTCTTGCTGGCCCC	Sigma-Aldrich	N/A
mce3E-CR-F primer: GGGAAGGTTGGCGTATTCGCCGCG	Sigma-Aldrich	N/A
mce3E-CR-R primer: AAACCGCGGCGAATACGCCAACCT	Sigma-Aldrich	N/A
fadA2-CR-F primer: GGGAAACGGACTGACCAGGTCGTC	Sigma-Aldrich	N/A
fadA2-CR-R primer: AAACGACGACCTGGTCAGTCCGTT	Sigma-Aldrich	N/A
pknG-CR-F primer: GGGAACGGGACGTCCACAGTTCCA	Sigma-Aldrich	N/A
pknG-CR-R primer: AAACTGGAACTGTGGACGTCCCGT	Sigma-Aldrich	N/A
echA21-CR-F primer: GGGA AACACCTCGGGCATCTCCGA	Sigma-Aldrich	N/A
echA21-CR-R primer: AAAC TCGGAGATGCCCGAGGTGTT	Sigma-Aldrich	N/A
Rv1463-CR-F primer: GGGAACCTCGACGGGATATTGCATGG	Sigma-Aldrich	N/A
Rv1463-CR-R primer: AAACCCATGCAATATCCCGTCGAGGT	Sigma-Aldrich	N/A
ptpA-CR-F primer: GGGAGTTCCCGAGCGTGGGTCGAATGACC	Sigma-Aldrich	N/A
ptpA-CR-R primer: AAACGGTCATTCGACCCACGCTCGGGAAC	Sigma-Aldrich	N/A
gnd2-CR-F primer: GGGAAGGATGTTCAATCCCTCCGC	Sigma-Aldrich	N/A
gnd2-CR-R primer: AAACGCGGAGGGATTGAACATCCT	Sigma-Aldrich	N/A
Rv3224-CR-F primer: GGGAGCAGGATCGGCGGGGACAGCG	Sigma-Aldrich	N/A
Rv3224-CR-R primer: AAACCGCTGTCCCCGCCGATCCTGC	Sigma-Aldrich	N/A
